# A Cas9-mediated adenosine transient reporter enables enrichment of ABE-targeted cells

**DOI:** 10.1186/s12915-020-00929-7

**Published:** 2020-12-14

**Authors:** Nicholas Brookhouser, Toan Nguyen, Stefan J. Tekel, Kylie Standage-Beier, Xiao Wang, David A. Brafman

**Affiliations:** 1 School of Biological and Health Systems Engineering, Arizona State University, 501 E. Tyler Mall, ECG 334A, Tempe, AZ 85287 USA; 2 Graduate Program in Clinical Translational Sciences, University of Arizona College of Medicine-Phoenix, Phoenix, AZ 85004 USA; 3 Molecular and Cellular Biology Graduate Program, Arizona State University, Tempe, AZ 85287 USA

**Keywords:** CRISPR, Genome modification, Base editor, Human pluripotent stem cells, Multiplexing

## Abstract

**Background:**

Adenine base editors (ABE) enable single nucleotide modifications without the need for double-stranded DNA breaks (DSBs) induced by conventional CRIPSR/Cas9-based approaches. However, most approaches that employ ABEs require inefficient downstream technologies to identify desired targeted mutations within large populations of manipulated cells. In this study, we developed a fluorescence-based method, named “Cas9-mediated adenosine transient reporter for editing enrichment” (CasMAs-TREE; herein abbreviated XMAS-TREE), to facilitate the real-time identification of base-edited cell populations.

**Results:**

To establish a fluorescent-based assay able to detect ABE activity within a cell in real time, we designed a construct encoding a mCherry fluorescent protein followed by a stop codon (TGA) preceding the coding sequence for a green fluorescent protein (GFP), allowing translational readthrough and expression of GFP after A-to-G conversion of the codon to “TGG.” At several independent loci, we demonstrate that XMAS-TREE can be used for the highly efficient purification of targeted cells. Moreover, we demonstrate that XMAS-TREE can be employed in the context of multiplexed editing strategies to simultaneous modify several genomic loci. In addition, we employ XMAS-TREE to efficiently edit human pluripotent stem cells (hPSCs), a cell type traditionally resistant to genetic modification. Furthermore, we utilize XMAS-TREE to generate clonal isogenic hPSCs at target sites not editable using well-established reporter of transfection (RoT)-based strategies.

**Conclusion:**

We established a method to detect adenosine base-editing activity within a cell, which increases the efficiency of editing at multiple genomic locations through an enrichment of edited cells. In the future, XMAS-TREE will greatly accelerate the application of ABEs in biomedical research.

## Background

CRISPR/Cas9-based approaches have allowed for genome engineering in a variety of cells, tissues, and organisms [1–3]. Conventional RNA-programmable Cas9-endonucleases introduce DNA double-stranded breaks (DSBs) at precise chromosomal locations. Typically, these DSBs are repaired by non-homologous end joining (NHEJ) which leads to gene disruption through the insertion or deletion (indels) of DNA sequences [2]. In the presence of an exogenous DNA template, these DSBs can instead be repaired by homology-directed repair (HDR) allowing for modification of the genome at single nucleotide resolution. However, HDR is not only less efficient than NHEJ but also requires active cell division [4]. As such, the use of HDR-based approaches to modify individual base pairs has been difficult to achieve, especially in cells which are resistant to genomic modification [4].

Because of the limitations of CRISPR/Cas9 methods, engineered base editors (BEs) have emerged as a powerful technology to introduce single base pair changes without the need for the introduction of DSBs and the use of inefficient HDR [2, 5]. To date, two types of base editors have been developed—cytosine base editors (CBEs) which facilitate the conversion of C-to-T (or G-to-A) conversions and adenine base editors (ABEs) which mediate the change of A-to-G (or T-to-C) [2]. While both editors utilize a nicking Cas9 variant (Cas9^D10A^) for genomic targeting, CBEs employ an APOBEC1 cytosine deaminase whereas ABEs utilize an engineered TadA adenosine deaminase to introduce their respective nucleotide changes [6]. Both classes of BEs have been used in a variety of applications, including the investigation of developmentally relevant signaling pathways, modulation of gene regulatory networks, interrogation of disease-causing point mutations, generation of animal model systems, and in vivo correction of pathogenic mutations in somatic cells [2, 5]. Importantly, these BEs do not require the use of potentially harmful DSBs and allow for single base pair modifications with a lower incidence of indels and off-target genome modification than traditional CRISPR/Cas9 editing methodologies [2, 5, 6].

Despite the advantages of BEs, the identification of edited cell populations requires the use of end-point sequencing assays. In addition, it has been shown that various parameters including editor expression, nuclease activity, target site accessibility, and sgRNA pairing can limit the efficiency of BE-mediated genome modification [6–10]. In particular, this could limit the use of BEs to modify endogenous target loci that are resistant to editing as it might necessitate the laborious screening of a large number of clonal lines to identify cells with the desired mutation. Consequently, real-time methods that can identify and enrich for base-edited cell populations are needed. Previously, various fluorescence-based assays have been developed to report on CRISPR/Cas9-induced HDR [11, 12]. Leveraging this work, several groups, including our own, have developed fluorescence-based strategies to report on CBE activity within a cell and to purify modified cell populations [6, 10, 13, 14]. However, the development of similar ABE reporters has been much more limited [6, 15]. For example, Kattie and colleagues recently described the generation of an activatable GFP-based system to report on ABE kinetics within a cell [6]. However, this reporter requires viral transduction possibly resulting in integration of the reporter into the genome of the targeted cells, which might limit their downstream use. In addition, this system was only shown to measure ABE enzyme activity and was not employed to enrich for ABE editing events at target loci.

In this study, we established a method entitled a Cas9-mediated adenosine transient reporter for editing enrichment (CasMAs-TREE; herein abbreviated XMAS-TREE) to detect ABE activity within a cell. In turn, we demonstrate at several independent loci that XMAS-TREE can be used to rapidly identify and purify modified cell populations. In addition, we show that XMAS-TREE can be used in concert with multiplex editing schemes to efficiently edit several independent loci. Critically, we use XMAS-TREE to edit human pluripotent stem cells (hPSCs), a cell type refractory to traditional gene editing approaches. In particular, we show that XMAS-TREE allows for the efficient generation of clonal isogenic hPSCs at loci not editable using typical reporter of transfection (RoT)-based enrichment techniques. Collectively, XMAS-TREE is an easily implemented method that will greatly facilitate the use of ABEs in downstream basic biomedical science and translational applications.

## Results

### Development of a fluorescent reporter for Cas9-mediated adenosine base editing

**Fig. 1:**
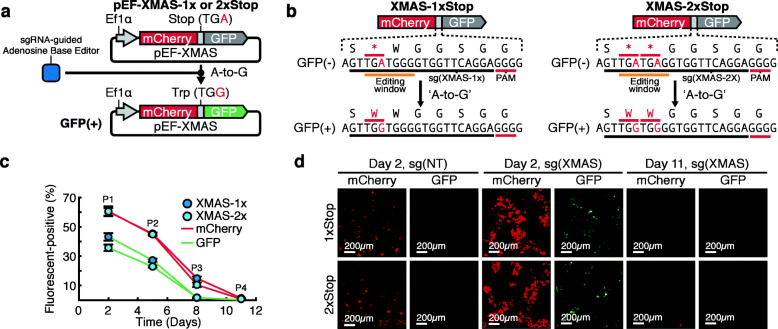
A fluorescent reporter system for real-time measurement of adenosine base-editing activity. **a** The XMAS-TREE reporter vector consists of a human EF1α promoter driving expression of an mCherry cassette followed by a stop codon (TGA), and a GFP cassette. Targeting pEF-XMAS with an adenine base editor and sg(XMAS) will result in an A-to-G conversion, enabling expression of the downstream GFP reporter. **b** Two versions of pEF-XMAS-TREE plasmid were designed, one with a single stop codon (XMAS-1xStop) and another with two stop codons (XMAS-2xStop), preceding the coding sequence for GFP. The protospacer sequence (underlined black) for the sgRNA, sg(XMAS), targeting the TGA codon (underlined red) resulting in an A-to-G conversion to TGG and the corresponding amino acid change to tryptophan. The PAM sequence (underlined red) was placed to position the base editing window (underlined orange) around the target nucleotides. **c** Flow cytometry and **d** fluorescence microscopy analysis of HEK293 cells at various time points after transfection with pEF-XMAS-1xStop (top panels) or pEF-XMAS-2xStop (bottom panels), pCMV-ABEmax, and sg(XMAS)

Conventional enrichment strategies that are used by others, such as co-transfection with a fluorescent protein, only report on the efficiency of plasmid delivery to a cell. Along similar lines, as we have previously shown with cytosine base editors (CBEs), reporters of expression in which a fluorescent protein is expressed along with the base editor do not directly report on base-editing activating within a cell [16–21]. To determine if the same was true with adenosine base-editing approaches, we transfected HEK293 cells with a reporter plasmid (mCherry), an adenine base editor (ABEmax; pCMV-ABEmax), and a sgRNA for a genomic target site [sg(TS)] [22]. This analysis revealed limited correlation between transfection efficiency (percentage of mCherry-positive cells) and editing efficiency (percentage of A-to-G conversion at target nucleotide) (Additional File 1: Fig. S1). Along similar lines, previous work has shown that other factors including intracellular formation of Cas9/sgRNA complexes, cell cycle, chromatin accessibility, local epigenetic modifications, and sequence context influence editing efficiency [23]. To that end, we sought to leverage our experience developing fluorescent reporters of editing activity to enable XMAS-TREE [16, 21]. To establish a fluorescent assay to detect ABE activity within a cell, we engineered a construct encoding a mCherry fluorescent protein followed by a stop codon (TGA) immediately preceding the coding sequence for a green fluorescent protein (GFP). Consequently, the A-to-G conversion of that codon to “TGG” (encoding tryptophan) will enable translational readthrough and expression of GFP (Fig. 1a). The protospacer is preceded by a T2A self-cleavage peptide, allowing for separation of mCherry and GFP (Fig. 1b). The protospacer falls within a flexible glycine-serine sequence. To determine the utility of this fluorescent-based construct to report on ABE activity, we assembled a vector with a human EF1α promoter to drive expression of the fluorescent reporters (pEF-XMAS; Fig. 1a). In addition, we engineered two versions of this vector, one with a single stop codon (pEF-XMAS-1xStop) and another with two stop codons (pEF-XMAS-2xStop; Fig. 1b). We speculated that A-to-G conversion of two stop codons within the editing window would provide a higher degree of stringency with respect to reporting on base-editing activity within a cell. In addition, we designed a sgRNA vector [sg(XMAS)] that would direct the ABE to the target “TGA” resulting in an A-to-G conversion and allow for subsequent translation of the downstream GFP cassette. Next, HEK293 cells were co-transfected with pEF-XMAS, pCMV-ABEmax, and sg(XMAS) or a control non-targeting sgRNA [sg(NT)]. Flow cytometry (Fig. 1c) and flourescence microscopy (Fig. 1d) revealed that targeting pEF-XMAS with sg(XMAS) resulted in the generation of mCherry/GFP double-positive cells, suggesting A-to-G base editing in the target codons allowing for GFP expression. Conversely, targeting pEF-XMAS-1xStop or pEF-XMAS-2xStop with sg(NT) did not result in the generation of any GFP-positive cells (Additional File 2: Fig. S2). Despite similarities in transfection efficiency between pEF-XMAS-1xStop and pEF-XMAS-2xStop (as measured by percentage of mCherry-positive cells), the percentage of GFP-positive cells was significantly lower in sg(XMAS) targeted cells transfected with pEF-XMAS-2xStop, suggesting that a higher level of base-editing activity was necessary for the activation of GFP expression with the 2xStop plasmid. Interestingly, a significant percentage of cells that were mCherry-positive were not GFP-positive, verifying that the reporter of transfection (mCherry) does not report on base-editing activity within a cell (Additional File 1: Fig. S1). Finally, we wanted to demonstrate that the fluorescent output associated with the XMAS-TREE reporter was transient. As such, we measured the long-term fluorescence of cells transfected with pEF-XMAS and targeted with sg(XMAS). Indeed, analysis of these cells by flow cytometry (Fig. 1c) and fluorescence microscopy (Fig. 1d) revealed no long-term detectable fluorescent signal, confirming that the XMAS-TREE fluorescent output was transient. Collectively, this data establishes that editing of the XMAS-TREE plasmid provides a transient fluorescent reporter for base-editing activity within a cell.

### XMAS-TREE allows for the identification and isolation of base-edited cell populations

**Fig. 2:**
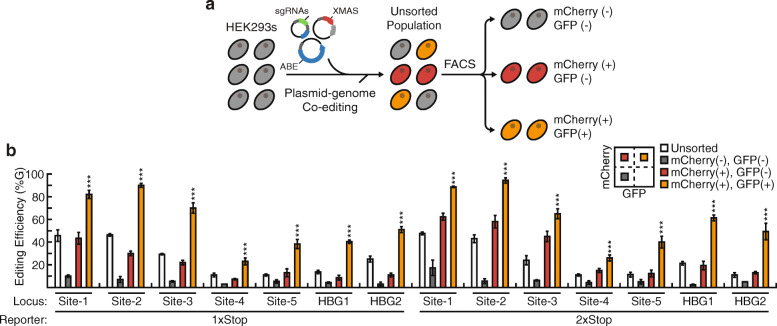
Identification and enrichment of base-edited cell populations using XMAS-TREE. **a** Schematic for identification and enrichment of adenosine base edited cells using XMAS-TREE. Cells are transfected with pEF-XMAS, pCMV-ABEmax, and pTS-sgRNA vectors. Post-transfection, flow cytometry is used to sort cell populations into reporter-positive and reporter-negative populations based upon mCherry and GFP expression levels. **b** Quantification of base-editing efficiency at various genomic loci in unsorted (white bar), mCherry-negative/GFP-negative (gray bar), mCherry-positive/GFP-negative (red bar), and mCherry/GFP double-positive (orange bar) isolated cells using XMAS-TREE-based methods. One-way ANOVA with Tukey post hoc test was performed. Statistical significance is only shown for the comparisons made between cells positive for the transfection reporter but not the editing reporter (i.e., mCherry-positive/GFP-negative) and cells positive for both (i.e., mCherry/GFP double-positive); *p < 0.05, **p < 0.01, ***p < 0.001. *n* = 3

Next, we wanted to demonstrate the utility of XMAS-TREE for the identification and isolation of cells in which targeted genomic adenosine base editing had occurred. To facilitate this, we designed a dual-targeting sgRNA (pDT-sgRNA) vector that contains both sg(XMAS) and a guide matching an endogenous target site, sg(TS). Additionally, the pDT-sgRNA vector was designed to allow for the straightforward cloning of new target sites via BbsI restriction enzyme digestion and ligation of sg(TS) oligonucleotides. We designed pDT-sgRNA vectors with sequences targeting five genomic loci (Sites 1–5) as well as the promoter of the γ-globin genes *HBG1* and *HBG2*. To utilize XMAS-TREE for enrichment of cells that have been edited at a specific genomic location, we co-transfected these pDT-sgRNA vectors with pEF-XMAS-1xStop or 2xStop and pCMV-ABE into HEK293 cells (Fig. 2a). Flow cytometry was then used to isolate fluorescent cell populations and Sanger sequencing was performed on the targeted genomic sites in isolated populations (Fig. 2a). As expected, mCherry/GFP double-positive cells were enriched for edited cells when compared to double-negative cell populations (Fig. 2b). Importantly, the transfection marker mCherry-positive population had significantly reduced editing compared to the based editing reporter positive GFP-positive population. This demonstrates the benefit of utilizing a real-time reporter of base editing (Fig. 2b). In addition, we wanted to compare the editing efficiency of XMAS-TREE compared to conventional reporters of transfection (RoT) strategies used by others [17–20]. As such, we co-transfected HEK293 cells with a reporter plasmid (pEF-mCherry), an adenine base editor (pCMV-ABEmax), and a sgRNA for various genomic target sites [sg(TS)]. Flow cytometry was then used to sort mCherry-positive cell populations (RoT), and Sanger sequencing was performed on the targeted genomic sites. This analysis revealed that across all target sites the mCherry/GFP double-positive cells isolated using XMAS-TREE with the 2xStop vector had a significantly higher frequency of base editing than mCherry-positive cells isolated using traditional RoT approaches (Additional File 3: Fig. S3). By comparison, in only 5 of the 7 target sites, the mCherry/GFP double-positive cells isolated using XMAS-TREE with the 1xStop vector had a significantly higher frequency of base editing than mCherry-positive cells isolated using conventional RoT strategies (Additional File 3: Fig. S3). Interestingly, XMAS-TREE demonstrated the largest relative improvement in base-editing efficiency compared to RoT approaches at loci that were more difficult to edit (e.g., Site-4, Site-5, HBG1, HBG2). Nonetheless, these results confirm that XMAS-TREE could be used to identify and enrich for adenosine base-edited cell populations at a variety of genomic target sites.

Because our early analysis suggested that a higher level of base-editing activity was necessary for activation of the GFP expression with the 2xStop plasmid when compared to the 1xStop plasmid, we speculated that using the 2xStop vector versus the 1xStop vector would result in a higher degree of base editing at target loci. However, quantification of base-editing efficiency in mCherry/GFP double-positive cells isolated using XMAS-TREE based targeting revealed no difference between the use of 1xStop and 2xStop reporting vector (Additional File 4: Fig. S4). In addition, this analysis revealed that there was no difference in the level of bystander editing (i.e., editing at an A other that the target A within the editing window) between cells isolated using the 1xStop versus 2xStop reporting vector (Additional File 5: Fig. S5). Together, this data indicates that the 2xStop plasmid does not provide a higher level of stringency or processivity as it relates to its ability to enrich for edited cell populations. Finally, we examined if XMAS-TREE based enrichment led to a relative increase in bystander editing in sorted cell versus unsorted populations (Additional File 6: Fig. S6a). As expected, for a particular site, the absolute percentage of bystander edits increased proportionally with the level of editing at the target nucleotide. Consequently, the frequency of bystander editing is higher in the XMAS-TREE sorted mCherry/GFP double-positive cells compared to that in the unsorted populations because the frequency of on-target editing is concomitantly higher in the mCherry/GFP double-positive cells than the unsorted cells. However, our analysis reveals that the relative ratio of on-target to bystander editing is similar between the two populations.

### XMAS-TREE enables efficient multiplex base editing at genomic loci

**Fig. 3:**
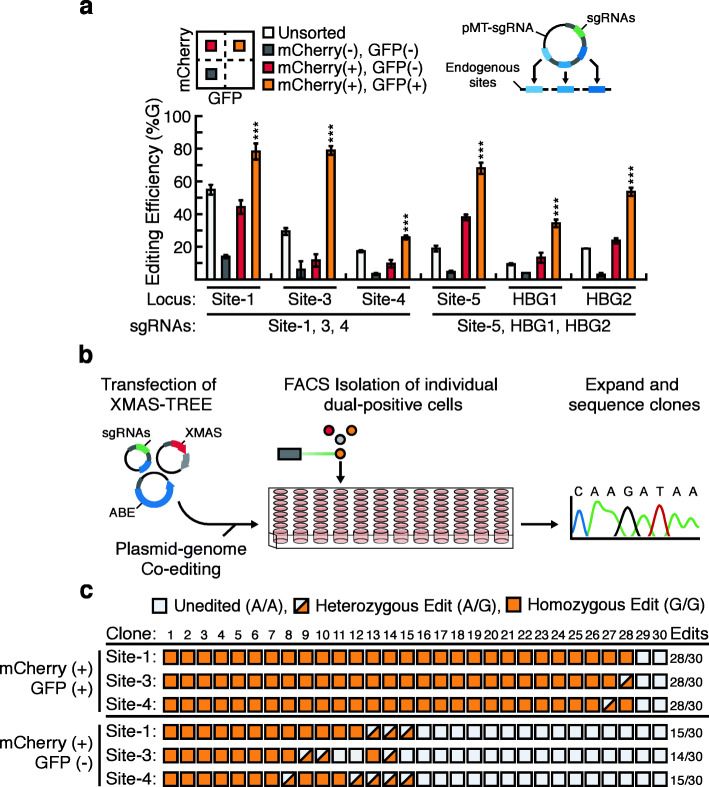
XMAS-TREE enables highly efficient multiplex adenosine base editing in HEK293 cells. **a** Cells were transfected with pEF-XMAS, pCMV-ABEmax, and a pMT-sgRNA that simultaneously targeted Site-1/Site-3/Site-4 or Site-5/HBG1/HBG2. Base editing was quantified in unsorted as well as reporter-positive and reporter-negative cell populations. One-way ANOVA with Tukey post hoc test was performed. Statistical significance is only shown for the comparisons made between cells positive for the transfection reporter but not the editing reporter (i.e., mCherry-positive/GFP-negative) and cells positive for both (i.e., mCherry/GFP double positive); *p < 0.05, **p < 0.01, ***p < 0.001. *n* = 3. **b** Schematic for employing XMAS-TREE for generation of clonal lines that have been simultaneously edited at multiple loci. HEK293 cells are co-transfected with pEF-XMAS, pCMV-ABEmax, and pMT-sgRNA. At 48 h post-transfection, single mCherry/GFP double-positive cells are sorted into 96-well plates. After expansion, target clones are identified by Sanger sequencing at the target sites. **c** Analysis of clonal editing efficiency at multiple independent genomic sites using XMAS-TREE. A total of 30 clones derived from the mCherry/GFP double-positive and mCherry-positive/GFP-negative cell populations were examined at each locus. White box indicates no editing observed a specified locus, half-filled box indicates mono-allelic targeting at the genomic site, and full box indicates bi-allelic editing at the target locus

We further evaluated XMAS-TREE to determine if it could be used for multiplexed genome editing. To that end, we generated a multi-targeting vector (pMT-sgRNA) that contains sg(TREE) as well as sgRNAs for multiple genomic targets. More specifically, we generated two pMT-sgRNA vectors—one that would target Site-1/Site-3/Site-4 and another that would simultaneously edit Site-5/HBG1/HBG2. We employed XMAS-TREE to simultaneously target multiple genomic sites by co-transfecting HEK293 cells with pMT-sgRNA, pEF-XMAS, and pCMV-ABEmax. Reporter-positive and reporter-negative cells were isolated by flow cytometry and analyzed by Sanger sequencing at the targeted loci. Consistent with single locus targeting, mCherry/GFP double-positive cells displayed a significantly higher frequency of base editing at the target sites than editing levels that were observed in unsorted, mCherry-negative/GFP-negative, and mCherry-positive/GFP-negative cell populations (Fig. 3a). Importantly, there was no significant reduction in editing efficiency when XMAS-TREE was used to target these sites individually or a multiplexed fashion (Additional File 7: Fig. S7). Moreover, Sanger sequencing of the multiplex targeted genomic sites in cells isolated from XMAS-TREE and RoT approaches reveal that XMAS-TREE allowed for statistically significantly higher frequency of base editing than RoT approaches (Additional File 8: Fig. S8). Finally, our analysis revealed similarly to when a single site is targeted that multiplex targeting using XMAS-TREE did not result in a substantial increase in the relative levels of bystander editing in isolated cell populations (Fig. 6b).

Our initial analysis of bulk sorted mCherry/GFP double-positive cells suggested that multiplexed editing with XMAS-TREE resulted in a large percentage of cells that had been simultaneously edited at multiple loci. To verify this observation, we utilized XMAS-TREE for the clonal isolation of base-edited populations (Fig. 3b). Briefly, we co-transfected HEK293 cells with pEF-XMAS, pCMV-ABEmax, and a pMT-sgRNA designed to simultaneously target genomic Site-1/Site-3/Site-4. Single mCherry-positive/GFP-positive cells were sorted into a 96-well plate and expanded prior to analysis. Genomic DNA was isolated from clonal populations and the multiplexed genomic sites were subject to Sanger sequencing after PCR amplification (Additional File 9: Fig. S9). Remarkably, this analysis revealed that greater than 90% of the clones isolated had been edited, with 26 out of the 30 clones having biallelic conversions at all three genomic loci (Fig. 3c, top panel). By comparison, analysis of clones isolated from single cells only positive for the transfection reporter (mCherry-positive/GFP-negative) displayed significantly lower editing efficiency, with only 7 out of the 30 clones having biallelic conversions at all three genomic loci (Fig. 3c, bottom panel).

Next, we wanted to evaluate the level of bystander editing in clonal populations isolated using BIG-TREE strategies. This analysis revealed a number of clones in which at simultaneously at genomic Site 1 and Site 4 modification only occurred at the target A and not at any other A’s within the editing window (Additional File 10: Fig. S10). More specifically, of the 26 clones that had biallelic edits at all 3 sites, 6 clones were free from bystander edits at both Site 1 and Site 4 (clones 5, 7, 11, 13, 15, 14), 5 clones were free from bystander edits at Site 1 only (clones 2, 3, 10, 16, 23), and 9 clones were free from bystander edits at Site 4 only (clones 1, 6, 12, 14, 17, 21, 22, 26, 27). However, it should be noted that we did not identify any clones at which genomic site 3 that modification at only the target A occurred. Instead, we speculate that because another A occurs 1 nucleotide away from the target A still within the optimal editing window that such exclusive modification is likely a difficult to achieve event that will necessitate the use of base editors that provide for exclusive editing of the target A free from bystander editing at neighboring nucleotides [23].

Lastly, we wanted to determine if XMAS-TREE increased A-to-G conversion at off-target loci. Therefore, in several clones that had biallelic edits at all three target sites, we performed off-target analysis at the top predicted sites for sg(XMAS) as well as the sgRNAs used to target Site-1/Site-3/Site-4. At all of the off-target sites analyzed, we did not observe substantial A-to-G edits at these off-target loci (Additional File 11: Fig. S11). In addition, indels were not observed at any of the off-target sites in the clones analyzed. Collectively, these results demonstrate the broad utility of XMAS-TREE to allow for the highly efficient, simultaneous editing of multiple independent loci.

### Highly efficient editing of human pluripotent stem cells (hPSCs) using XMAS-TREE

**Fig. 4:**
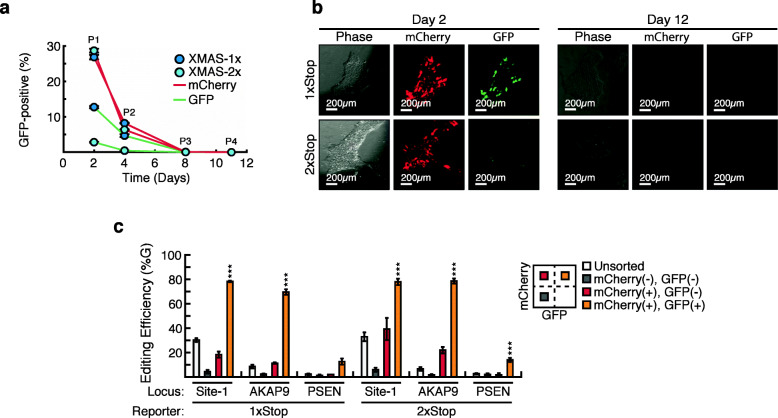
XMAS-TREE can be employed for the highly efficient base editing of human pluripotent stem cells (hPSCs). **a** Flow cytometry and **b** fluorescence microscopy analysis of hPSCs at various time points after transfection with pEF-XMAS-1xStop (top panels) or pEF-XMAS-2xStop (bottom panels), pEF-ABEmax, and sg(XMAS). **c** Quantification of base-editing efficiency at various genomic loci in unsorted (white bar), mCherry-negative/GFP-negative (gray bar), mCherry-positive/GFP-negative (red bar), and mCherry/GFP double-positive (orange bar) isolated hPSCs using XMAS-TREE-based methods. One-way ANOVA with Tukey post hoc test was performed. Statistical significance is only shown for the comparisons made between cells positive for the transfection reporter but not the editing reporter (i.e., mCherry-positive/GFP-negative) and cells positive for both (i.e., mCherry/GFP double positive); *p < 0.05, **p < 0.01, ***p < 0.001. *n* = 3

Traditional CRISPR-based approaches to modify single base pairs in hPSCs suffer from extremely low efficiencies [24–27]. Therefore, we wanted to determine if XMAS-TREE could be utilized to efficiently mediate A-to-G conversions at specific loci in hPSCs. To confirm that the XMAS reporter was functioning in hPSCs, we transfected hPSCs with pEF-XMAS1xStop/2xStop, pEF-ABEmax, and sg(XMAS) or sg(NT). Similar to our experiments with HEK293 cells, fluorescence microscopy (Fig. 4a) and flow cytometry (Fig. 4b) with sg(XMAS), but not with sg(NT) (Additional File 12: Fig. S12), resulted in the generation of mCherry/GFP double-positive cells, indicative of adenosine base editing of the pEF-XMAS reporter plasmid. Additionally, this analysis revealed that the proportion of cells that were positive for the base-editing reporter (GFP) relative to the transfection reporter (mCherry) were markedly reduced in hPSCs, consistent with reports that hPSCs are recalcitrant to genomic modification [24–27]. In this vein, these results suggest that purifying hPSC populations solely with a reporter of transfection (mCherry) would significantly dilute out cells with targeted genomic base edits. In addition, the level of base editing of the 2xStop plasmid was significantly lower that than observed with the 1xStop, suggesting that the 2xStop plasmid provides a higher degree of stringency in identifying base-edited populations in hPSCs. Finally, flow cytometry (Fig. 4b) and fluorescence analysis (Fig. 4c) demonstrated that there was no detectable mCherry or GFP signal after 2 weeks of culture, confirming that the fluorescent signal associated with the XMAS-TREE reporter was transient in hPSCs.

Since we established pEF-XMAS reports on functional base editing in hPSCs, we wanted to determine if XMAS-TREE could be employed to enrich for cells with single-base pair edits at target loci in hPSCs. In this regard, we co-transfected hPSCs with pEF-XMAS1xStop/2xStop and pEF-ABEmax along with a pDT-sgRNA targeting genomic Site-1 or single base pair changes in AKAP9 and PSEN1 that have been previously associated with increased risks of developing Alzheimer’s disease (AD) [28, 29]. In turn, flow cytometry was used to purify reporter-positive and reporter-negative cell populations and Sanger sequencing was performed on the targeted genomic locations in isolated populations. This analysis demonstrated that mCherry/GFP double-positive cells displayed a statistically significant increase in editing efficiency at the target loci when compared to other populations analyzed (Fig. 4d). In fact, in the more difficult to edit loci, AKAP9 and PSEN1, editing was virtually absent in populations not positive for our base-editing reporter (GFP). Together, these results demonstrated that XMAS-TREE can be used for the isolation of base-edited hPSC populations.

Although our previous analysis with HEK293 cells showed no difference in editing efficiency between cells isolated using the 1xStop and 2xStop reporter plasmid, we speculated that the 2xStop construct might allow for greater enrichment in hPSCs, which are typically recalcitrant to gene editing. However, quantification of base editing at target loci revealed no difference in editing efficiency between mCherry/GFP double-positive cells isolated using the 1xStop or 2xStop reporting vector (Additional File 13: Fig. S13). As such, this data confirms that the 2xStop plasmid does not allow for greater enrichment of edited populations, even when used in the context of difficult to edit cells such as hPSCs. However, we did observe less leaky GFP expression when using the 2xStop reporter with sg(NT), which may be useful in applications where low background expression is desirable (Additional File 2: Fig. S2, Additional File 12: Fig. S12).

### XMAS-TREE enables highly efficient generation of clonal isogenic hPSC lines

**Fig. 5:**
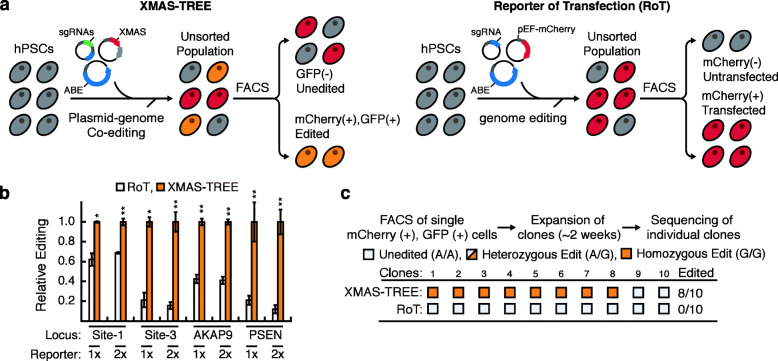
Highly efficient generation of clonal isogenic hPSC lines using XMAS-TREE. **a** Schematic for enrichment of adenosine base-edited cells using XMAS-TREE and reporter of transfection (RoT)-based approaches. **b** Quantification of relative base editing in mCherry-positive cells isolated using RoT and mCherry/GFP double-positive cells isolated using XMAS-TREE. Student’s *t* test; *p < 0.05, **p < 0.01, ***p < 0.001. *n* = 3. **c** Analysis of clonal editing efficiency in hPSCs that were targeted at the Site-3 locus using XMAS-TREE- or RoT-based methods

We next wanted to compare the editing efficiency enabled by XMAS-TREE compared to conventional reporters of transfection (RoT). Accordingly, we co-transfected hPSCs with a reporter plasmid (pEF-mCherry), an adenine base editor (pEF-ABEmax), and a sgRNA for various genomic target sites [sg(TS)] (Fig. 5a). Flow cytometry was then used to sort mCherry-positive cell populations (RoT), and Sanger sequencing was performed on the targeted genomic sites. This analysis revealed that across all targeted sites, mCherry/GFP double-positive cells isolated using XMAS-TREE had a significantly higher frequency of base editing than mCherry-positive cells isolated using traditional RoT approaches (Fig. 5b). In fact, several targeted loci (i.e., Site-3, PSEN) displayed undetectable levels of editing when traditional RoT approaches were applied (Additional File 14: Fig. S14). We then wanted to directly compare the efficiency by which XMAS-TREE and RoT-based methods could be utilized to generate clonal isogenic lines modified at these difficult to edit sites. To this end, we transfected hPSCs with pEF-XMAS, pEF-ABEmax, and pDT-sgRNA containing a sgRNA to target genomic Site-3. Single mCherry/GFP double-positive cells were sorted into 96-well plates, expanded, and subjected to Sanger sequencing. Of the 10 clones analyzed, 80% had a homozygous A-to-G edit at the genomic Site-3 locus (Fig. 5c). Importantly, indels were not identified in any of the clones at the target site. For comparison to a more conventional RoT approach to generate isogenic lines, this same hPSC line was transfected with a plasmid in which the base editor (ABEmax) was co-transfected with a pEF-mCherry vector as well as the same sgRNA for the Site-3 locus. After 48 h post-transfection, single GFP-positive cells were sorted into 96-well plates. Clonal lines were then passaged, expanded, and subjected to Sanger sequencing at the targeted locus. Notably, analysis of 10 clonal lines revealed that this RoT-based approach did not result in generation of a single isogenic clone at the target site (Fig. 5c). This ability of XMAS-TREE to generate isogenic clonal lines at sites that did not display significant editing in bulk RoT approaches was also confirmed at the PSEN locus (Additional File 15: Fig. S15). In sum, these results demonstrate that XMAS-TREE can not only provide for a higher level of enrichment of base-edited cell populations compared to RoT approaches, but also can allow for the generation of isogenic lines at genomic loci that are not achievable with conventional RoT methods.

Lastly, one of the limitations of most base editor techniques, irrespective if any enrichment strategies that are employed, is the induction of bystander edits at neighboring nucleotides within the editing window. To that end, we examined the level of bystander editing in these clonal populations. At Site-3, all clonal populations with an edit at the target A had a corresponding bystander edit at the neighboring A (Additional File 16: Fig. S16). By comparison, at the more difficult to edit PSEN 1 locus, one of the biallelic edited clones and two of the monoallelic edited clones were free from bystander edits. This suggests that some loci might require the use of next generation base editors with narrow editing windows if editing at a bystander A is not acceptable (i.e., an allelic change that results in changes in the amino acid coding sequence).

### Multiplex editing of hPSCs using XMAS-TREE

**Fig. 6:**
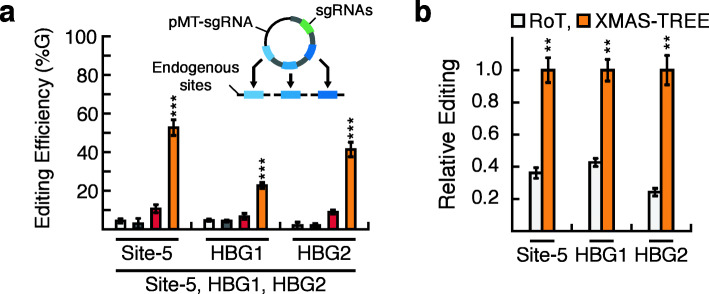
Simultaneous adenosine base editing of multiple target sites in hPSCs using XMAS-TREE. **a** HPSCs were co-transfected with pEF-XMAS, pEF-ABEmax, and a pMT-sgRNA that simultaneously targeted Site-5/HBG1/HBG2. Flow cytometry was used to sort reporter-positive and reporter-negative cell populations and base-editing was quantified at target loci. One-way ANOVA with Tukey post hoc test was performed. Statistical significance is only shown for the comparisons made between cells positive for the transfection reporter but not the editing reporter (i.e., mCherry-positive/GFP-negative) and cells positive for both (i.e., mCherry/GFP double positive); *p < 0.05, **p < 0.01, ***p < 0.001. *n* = 3. **b** Quantification of relative base editing in mCherry-positive and mCherry/GFP double-positive cells isolated using RoT and XMAS-TREE, respectively. Student’s *t* test; *p < 0.05, **p < 0.01, ***p < 0.001. *n* = 3

Lastly, we wanted to establish that XMAS-TREE could allow for multiplexed genome modification in hPSCs. HPSCs were co-transfected with pEF-XMAS, pEF-ABEmax, and a pMT-sgRNA with sgRNAs targeting Site-5, *HBG1*, and *HBG2*. Similar to our results obtained with HEK293 cells, mCherry/GFP double-positive cells had a statistically significant higher level of base editing at all three target sites when compared to those in unsorted, mCherry-negative/GFP-negative, and mCherry-positive/GFP-negative cell populations (Fig. 6a). In addition, direct comparison of multiplex editing using XMAS-TREE and RoT approaches demonstrated that XMAS-TREE allowed for a statistically significant higher level of base editing than by RoT-based methods (Fig. 6b). Finally, our analysis revealed that the relative ratio of on-target to bystander editing at all three loci is similar between mCherry/GFP double-positive and unsorted cell populations (Additional File 17: Fig. S17). This suggests that similar to what was observed in HEK293 cells, XMAS-TREE-based enrichment strategies do not lead to a relative enrichment of bystander editing. Overall, this data demonstrates that XMAS-TREE enables efficient simultaneous editing of multiple loci in hPSCs.

Because our early analysis suggested that a higher level of base-editing activity was necessary for activation of the GFP expression with the 2xStop plasmid when compared to the 1xStop plasmid, we speculated that using the 2xStop vector versus the 1xStop vector would result in a higher degree of base editing at target loci. However, quantification of base-editing efficiency in mCherry/GFP double-positive cells isolated using XMAS-TREE-based targeting revealed no difference between the use of 1xStop and 2xStop reporting vector (Additional File 4: Fig. S4). In addition, this analysis revealed that there was no difference in the level of bystander editing (i.e., editing at an A other that the target A within the editing window) between cells isolated using the 1xStop versus 2xStop reporting vector (Additional File 5: Fig. S5). Together, this data indicates that the 2xStop plasmid does not provide a higher level of stringency or processivity as it relates to its ability to enrich for edited cell populations. Finally, we examined if XMAS-TREE-based enrichment led to a relative increase in bystander editing in sorted cell versus unsorted populations (Additional File 6: Fig. S6). As expected, for a particular site, the absolute percentage of bystander edits increased proportionally with the level of editing at the target nucleotide. Consequently, the frequency of bystander editing is higher in the XMAS-TREE sorted mCherry/GFP double-positive cells compared to that in the unsorted populations because the frequency of on-target editing is concomitantly higher in the mCherry/GFP double-positive cells that the unsorted cells. However, our analysis reveals that the relative ratio of on-target to bystander editing is similar between the two populations.

## Discussion

Together, CBEs and ABEs have the potential ability to modify up to 60% of the disease-causing point mutations [30]. That said, BEs can be used in the context of cellular models of human disease models to establish genotype-to-phenotype relationship associated with genetic risk factors, investigate disease mechanisms, and test therapeutic strategies. In our previous work, we describe the development of a transient reporter for editing enrichment (TREE) as a fluorescence-based assay to report on cytosine base-editing (CBE) activity within a single cell [16]. In this work, we develop an analogous reporter system, Cas9-mediated adenosine transient reporter for editing enrichment (CasMAs-TREE; XMAS-TREE) that allows for the real-time detection of adenosine base editing (ABE) for the identification and enrichment of base-edited cell populations. Notably, at several loci, XMAS-TREE allows for the targeted gene editing at efficiencies approaching 90%. As part of these efforts, we also utilized XMAS-TREE to enrich for cells that have been edited at several disease-relevant loci including those associated with sickle-cell anemia (i.e., HBG1, HBG2) and Alzheimer’s disease (i.e., AKAP9, PSEN1) [28, 29, 31]. In addition, we demonstrate that XMAS-TREE can be used in the context of multiplex genome engineering strategies to facilitate simultaneous A-to-G (or T-to-C) conversions at several independent loci at the same efficiencies when single loci were targeted. Critically, the ability of XMAS-TREE to generate clonal lines that had been simultaneously edited at multiple loci will enable the facile generation of cell-based models of polygenetic diseases [32, 33]. Finally, we establish that the same XMAS-TREE-based methods can be applied in human pluripotent stem cells (hPSCs), a cell population in which gene editing technologies, including base editors and multiplex genome modification, have been challenging to implement [34]. In particular, we show that XMAS-TREE can facilitate the establishment of isogenic hPSC lines at loci that were not able to be modified using well-accepted reporter of transfection (RoT) methods. In fact, we show that at certain target sites that XMAS-TREE can allow for derivation of isogenic clonal populations with biallelic modification with 80% efficiency. Notably, all targeted clones were free from indels at all on-target sites. The clonal targeting efficiencies that we observe with XMAS-TREE in hPSCs are significantly higher than those previously reported with other CRISPR/Cas9-based methods, which are often in the single digits at most loci [24–27, 35]. In addition, the inefficiencies associated with these well-established methods make it difficult to achieve homozygous or multiplexed editing in hPSCs [34, 36–38].

Despite these enabling features of ABE genome modification approaches, several caveats exist with all base-editing approaches irrespective of any downstream cell enrichment strategies. One of the main concerns with all Cas9-based genome engineering strategies is the potential for nucleotide changes at off-target loci [39]. In this study, off-target analysis of clonal cell populations generated using XMAS-TREE enrichment strategies did not reveal any untargeted nucleotide conversions at the potential off-target genomic loci examined. In addition, none of the clones had indels at the on- or off-target loci. Another limitation of the specific ABE variants (ABE7.10 and ABEmax) employed in this study is that they can induce A-to-G (or T-to-C) conversions at bystander As (or Ts) within the editing window. In this regard, although numerous targeted clones isolated by XMAS-TREE had bystander edits, we did isolate numerous clones with modifications exclusively at the target nucleotide. In the future, base editor variants with a more stringent editing window or altered PAM specificities can be employed with XMAS-TREE to avoid undesirable bystander edits [19, 39–42].

We speculate that XMAS-TREE can be utilized in other applications not described in this study. For example, several groups have reported the generation of additional ABEs with non-NGG PAM specificities, narrower targeting windows, and reduced by-product formation [19, 41, 43]. Accordingly, future application of XMAS-TREE with these next-generation ABE variants will be straightforward. In addition, we anticipate that XMAS-TREE can be applied to induce alterations in target gene expression. More specifically, we previously described how CBEs can be used with other TREE-based strategies to generate gene knockout lines without the introduction of DSBs through in-frame conversion of “CAG” codon encoding for glutamine to a “TAG” pre-mature stop codon [21]. However, these approaches do not allow targeting for all genes and can be limited by the propensity of CBEs to induce genome-wide Cas9-independent off-target mutations [18, 22, 44, 45]. As an alternative, Wang and colleagues recently described an ABE-mediated strategy to induce gene knockout through modification of the ATG start codon to ACG or GTG [18]. Moving forward, XMAS-TREE can be utilized with such strategies to enrich for cell populations with targeted gene knockouts. We also anticipate that XMAS-TREE can be employed in the context of engineering in vivo disease models. More precisely, traditional CRISPR-based approaches are limited in their ability to generate animal models of disease that arise as a consequence of single nucleotide mutations [46, 47]. While base editors are able to generate such animal models, such base-editing-driven approaches suffer from low efficiency, similar to when employed for in vitro applications [46, 47]. In this vein, XMAS-TREE can greatly facilitate the use of base editors in generating animal models of human disease. Lastly, in vivo base-editing approaches have great potential in gene therapy applications, especially in post-mitotic cells in which CRISPR-based HDR is not achievable [2, 48–51]. However, when conventionally applied in vivo base editing does not allow for real-time analysis of editing efficiency or tracking of edited cell populations. In the future, we envision that XMAS TREE can be applied with such in vivo base-editing strategies to enable preclinical evaluation of potential therapeutic interventions.

## Conclusions

In summary, there are several features of XMAS-TREE-based methods that will enable extensive use by the research community. First, XMAS-TREE only requires the use of common lipid-based reagents for cell transduction. We envision that XMAS-TREE is compatible with other DNA delivery systems (i.e., electroporation) or expression methods (i.e., ribonucleoprotein complexes [RNP]) that have been utilized in other CRISPR/Cas9- and BE-based genome engineering strategies [52, 53]. In the future, XMAS-TREE-associated plasmids can also be easily cloned into non-integrating viral vectors to facilitate the development of in vivo gene editing methods [2]. Second, we have designed the sgRNA vectors to allow for the simple restriction enzyme-based cloning of new target sites. In this regard, we show that XMAS-TREE can allow for the highly efficient editing of a diverse set of loci across multiple cell lines. In the future, XMAS-TREE can be easily utilized in other animal, primary, immortalized, or non-mammalian cell types. In addition, because of the high editing efficiencies associated with XMAS-TREE, establishment of clonal lines with the targeted base pair edit does not require the screening of hundreds of clones, which is typical of other methods [24–27, 35]. Finally, although we do not demonstrate any differences between XMAS-1xStop and -2xStop plasmids in terms of editing efficiency in isolated cell populations, we speculate that in the future such plasmids might provide for discriminating enrichment strategies when used in the context of next generation base editors with narrower editing windows or altered PAM requirements. For example, a modified version of the XMAS-2xStop plasmid might allow for exclusion of editing events in which targeting at bystander nucleotides is not desired. In conclusion, these enabling features of XMAS-TREE will significantly enhance the use of ABE-based technologies in a variety of contexts and cell populations.

## Materials and methods

### HEK293 culture

All media components were purchased from ThermoFisher Scientific (Waltham, Massachusetts, USA) unless indicated otherwise. HEK293 cells were cultured on poly-L-ornithine (4 μg/mL; Sigma Aldrich, St. Louis MO, USA) coated plates using the following media: 1X high glucose DMEM, 10% (v/v) fetal bovine serum, 1% (v/v) l-glutamine penicillin/streptomycin. Every other day the culture medium was changed. Every 5 days, cells were enzymatically passaged with Accutase (ThermoFisher).

### Human pluripotent stem cell (hPSC) culture

HPSCs were maintained in mTeSR1 medium (Stemcell Technologies) on feeder-free Matrigel (Corning)-coated plates [21]. Subculture was performed every 3 days using Accutase (Life Technologies) in mTeSR1 medium supplemented with 5 μM Y-27632 (Tocris).

### Plasmid construction

PCR reactions for molecular cloning were performed using Phusion high fidelity DNA polymerase (New England Biolabs, NEB, Ipswich, MA, USA) using the manufacturer’s protocol. Restriction digests were performed according to the manufacturer’s instructions (NEB). Ligation reactions were carried out at 16 °C using T4 DNA ligase (NEB). Oligonucleotides were synthesized by Integrated DNA Technologies (Coralville, IA, USA). Plasmid cloning products were sequenced confirmed via Sanger sequencing (DNASU Sequencing Core, Genewiz). Plasmid sequences will be made available upon request.

For construction of the pEF-XMAS plasmids, pEF-XMAS-1xStop and pEF-XMAS-2xStop, the mCherry coding sequence was amplified with primers adding the 1x and 2x stop codon protospacers to the 3′ end of mCherry. The GFP coding sequence was amplified with primers removing GFP canonical “ATG” start codon. PCR products were purified using a PCR cleanup kit (Sigma Aldrich). Purified PCR products for mCherry-1x/2xStop were digested with EcoRI and SapI. The purified GFP PCR product was digested with SapI and HindIII. Both mCherry-1xStop or -2xStop and GFP digestion products were ligated into EcoRI and HindIII digested pEF-GFP (Addgene #11154).

For construction of the pDT-sgRNA vector, sgRNA cassettes encoding a U6 promoter and sg(XMAS-1x or 2x) were PCR amplified with primers adding EcoRI and SapI sites. A non-target guide cassette containing a U6 promoter and BbsI restriction sites, sg(NT), was amplified with primers adding SapI and XbaI sites. Purified PCR products were digested with corresponding restriction enzymes and ligated into EcoRI and XbaI digested pUC19 (NEB, GenBank Accession #: L09137). To clone guides targeting endogenous loci, 1 μg of oligonucleotide pairs was phosphorylated using T4 polynucleotide kinase (NEB) in 50 μl reactions at 37 °C for ≥ 1 h. Subsequently, oligonucleotides were duplexed by heating to reactions to 90 °C for 5 min on an aluminum heating block. Reactions were slowly returning the reaction to room temperature over approximately 1 h. pDT-sgRNA was digested using BbsI and dephosphorylated using Antarctic phosphatase (NEB). Guide duplexes were ligated in an equimolar ratio to digested pDT-sgRNA using T4 DNA ligase (NEB).

For construction of the pMT-sgRNA vector, two sgRNA cassettes targeting endogenous sites were amplified with primers adding 5′-HindIII and 3′-SapI sites and 5′-SapI and 3′-HindIII sites. PCR products were purified and digested with HindIII and SapI. pDT-sgRNA targeting an endogenous locus was digested with HindIII and dephosphorylated using Antarctic phosphatase (NEB). PCR products containing guide cassettes were ligated in an equimolar ratio to HindIII digested pDT-sgRNA using T4 DNA ligase (NEB).

All sgRNAs were synthesized as pairs of oligonucleotides as listed in Additional File 19: Fig. ST1.

For insertion of the EF1α promoter into pCMV-ABE (Addgene #107723) or pCMV-ABEmax (Addgene #112095), the EF1α promoter was PCR amplified from pEF-GFP (Addgene #11154) using primers adding SpeI and NotI sites. The amplified EF1α promoter was purified via PCR cleanup kit (Sigma Aldrich) and subsequently digested with SpeI and NotI (NEB). Two to 3 μg of pCMV-ABE or pCMV-ABEmax was digested with SpeI and NotI for ≥ 1 h at 37 °C. Digested ABE vector was then dephosphorylated by Antarctic phosphatase (NEB) for ≥ 1 h at 37 °C. The EF1α promoter was ligated in an equimolar ratio to digest pCMV-ABE or pCMV-ABEmax.

### HEK 293 transfections and clonal isolation

For transfections, HEK293 cells were transfected in 24-well tissue culture plates at 40% confluence with the following reagents per well: 300 ng pCMV-ABE, 100 ng sgRNA vector [sg(NT), sg(XMAS), pDT-sgRNA, or pMT-sgRNA], 100 ng pEF-XMAS, 0.75 uL Lipofectamine 3000 Transfection Reagent (ThermoFisher), and 1 uL P3000 reagent (Thermo Fisher). All cells were harvested for sorting and/or analysis 48 h post-transfection. For clonal isolation, single GFP+ cells were sorted (BD FASAria IIu) into a single well of a PLO-coated 96-well plate. Cells were expanded to a 24-well plate prior to analysis.

### HPSC transfections and clonal isolation

hPSCs were passaged onto Matrigel-coated 12-well plates with 5 μM Y-27632. Media was changed, and transfection was performed 24 h after passage. Nine hundred-nanogram base editor (pEF-ABEmax), 300 ng sgRNA [sg(NT), sg(XMAS), pDT-sgRNA, or pMT-sgRNA], and 300 ng pEF-XMAS were transfected per well using 4 μL Lipofectamine Stem transfection reagent (Life Technologies). Media was changed 24 h post-transfection. Cells were dissociated using Accutase 48 h post-transfection and passed through a 0.45-μm filter. Single GFP-positive hPSCs were FACS sorted (BD FACSAria IIu) into 96-well Matrigel-coated plates in mTeSR1 supplemented with CloneR (Stemcell Technologies), plates were immediately centrifuged at 100×*g* for 1 min and incubated at 37 °C. Media was changed 48 h post-sort with fresh mTeSR1 supplemented with CloneR. Ninety-six hours post-sort, media was changed to mTeSR1 without supplement and clonal hPSC colonies were expanded with fresh media changes daily until ready for subculture.

### Quantification of base-editing efficiency

To isolate genomic DNA from bulk transfections, cells were directly sorted into a 50 μL master mix consisting of 1X Phire Hot Start II DNA Polymerase (ThermoFisher), 1 μM forward primer, and 1 μM reverse primer. PCR was performed using the primers in Additional File 19: Table S2 and the PCR conditions listed in Additional File 20: Table S3. All products sizes were confirmed on a 1% agarose gel prior to Sanger sequencing. Amplicons were purified using the QIAquick PCR purification kit (Qiagen) according to manufacturer’s instructions prior to Sanger sequencing. PCR products were column purified and Sanger sequencing (Genewiz) was performed using the primers listed in Additional File 19: Table S2. EditR was used to analyze Sanger sequence chromatographs to assess bating editing efficiencies with the parameters listed in Additional File 21: Table S4 [54].

### Genotyping of clonal populations

For analysis of clonal populations, genomic DNA was prepared from expanded clones using the DNeasy kit (Qiagen) and PCR products were generated with Phusion High-Fidelity Polymerase (New England Biolabs). PCR was performed in a similar manner to that described for the bulk transfections.

### Off-target analysis

For the data presented in Additional File 11: Fig. S11, analysis was performed for the top three off-target loci for the indicated sgRNAs predicted in silico via CCTop using default parameters for *S. pyogenes* Cas9 against human genome reference sequence hg38 [55]. Determination of base editing at these off-target sites was performed using CLC Main (Qiagen) and aligning the Sanger sequencing of the sample to the protospacer sequencing of the wild type. The PCR primers used to analyze these off-target sites are presented in Additional File 22: Table S5.

### Fluorescence microscopy

Fluorescence microscopy was performed with a Nikon Ti-Eclipse inverted microscope with an LED-based Lumencor SOLA SE Light Engine using a Semrock band pass filter. GFP was visualized with an excitation at 472 nm and emission at 520 nm. mCherry was visualized with an excitation of 562 nm and emission at 641/75 nm.

### Flow cytometry analysis

Cells were dissociated with Accutase for 10 min at 37 °C, triturated, and passed through a 40-μm cell strainer. Cells were then washed twice with flow cytometry buffer (BD Biosciences) and resuspended at a maximum concentration of 5 × 10^6^ cells per 100 μL. Flow cytometry analysis was performed on an Attune NxT (Thermo Fisher Scientific). Flow cytometry files were analyzed using with FlowJo (FlowJo LLC, Ashland, OR, USA).

### Statistical analysis

Data are displayed as mean ± standard deviation (S.D), unless otherwise stated. Student’s *t* test was used to make pairwise comparisons. ANOVA statistical methods were used to make multiple comparisons.

## Supplementary information

**Additional file 1: Fig. S1.** Transfection efficiency is not predictive of editing efficiency. HEK293 cells were transfected with pEF-mCherry, pCMV-ABEmax, and sg(TS). Comparison of transfection efficiency (percentage of mCherry-positive cells) and editing efficiency (percentage of A-to-G conversion at target nucleotides) in unsorted cell populations targeted at various genomic loci.

**Additional file 2: Fig. S2.** Flow cytometry-based characterization of XMAS-TREE reporter. Representative flow cytometry plots of HEK293 cells transfected with pEF-XMAS-1xStop or pEF-XMAS-2xStop, pCMV-ABEmax, and sg(NT) or sg(XMAS).

**Additional file 3: Fig. S3.** Comparison of editing efficiency in HEK293 cells isolated using RoT and XMAS-TREE approaches. Quantification of relative base editing at target loci in mCherry-positive cells isolated using RoT and mCherry/GFP double positive cells isolated using XMAS-TREE. Student’s t-test; N.S. = not significant, * = *p* < 0.05, ** = *p* < 0.01. *n* = 3

**Additional file 4: Fig. S4.** Comparison of editing efficiency in HEK293 cells at target loci using the 1xStop and 2xStop reporting vector. Quantification of base editing efficiencies at target loci in mCherry/GFP double positive cell populations using XMAS-TREE based targeting with the 1xStop or 2xStop reporting vector. Student’s t-test, N.S. = not significant. n = 3

**Additional file 5: Fig. S5.** Analysis of bystander editing in base-edited HEK293 cell populations using XMAS-TREE. Distribution of bystander edits at target loci in mCherry/GFP double positive cell populations isolated using XMAS-TREE based strategies. Orange indicates target A within the editing window. Light grey indicates bystander A within the editing window.

**Additional file 6: Fig. S6.** Comparison of bystander editing in mCherry/GFP double positive and unsorted HEK293 cell populations using XMAS-TREE. Distribution of bystander edits at target loci in mCherry/GFP double positive and unsorted cell populations using XMAS-TREE based strategies in the context of **(a)** singleplex and **(b)** multiplex editing. Orange indicates target A within the editing window. Light grey indicates bystander A within the editing window. Bystander ratio was computed as the frequency of editing the bystander A divided by the percentage of editing at the target A. *P*-value given for Student’s t-test comparing bystander ratio in mCherry/GFP double positive versus unsorted cells at an indicated bystander A. *n* = 3

**Additional file 7: Fig. S7.** Comparison of XMAS-TREE editing efficiency in individual- or multiplexed-targeted genomic sites in HEK293 cells. Quantification of base editing efficiencies at targeted loci in mCherry/GFP double positive cell populations using XMAS-TREE-based targeting in a single or multiplexed manner. Student’s t-test; N.S. = not significant, * = *p* < 0.05. n = 3

**Additional file 8: Fig. S8.** Comparison of multiplexed editing efficiency in HEK293 cells isolated using RoT and XMAS-TREE approaches. Quantification of multiplexed base editing efficiencies at target loci in mCherry-positive cells isolated using RoT and mCherry-positive/GFP-positive cells isolated using XMAS-TREE. Student’s t-test; N.S. = not significant, * = p < 0.05, ** = p < 0.01. n = 3

**Additional file 9: Fig. S9.** Representative Sanger sequences from clonal HEK293 cells isolated using XMAS-TREE in a multiplexed manner. Sanger sequences from a representative clone isolated using XMAS-TREE that had homozygous edits at all three-target loci, Site-1/Site-3/Site-4.

**Additional file 10: Fig. S10.** Analysis of bystander editing in clonal multiplexed base-edited HEK293 cell populations using XMAS-TREE. Distribution of bystander edits at genomic Site-1/Site-3/Site-4 in clonal HEK293 cells that were generated via multiplexed base editing.

**Additional file 11: Fig. S11.** Off-target analysis of HEK293 clones generated using XMAS-TREE-based methods. Representative clonal lines were analyzed by Sanger sequencing at the top predicted off-target loci for the sgRNA sequences for **(a)** sg(XMAS), **(b)** sg(Site-1), **(c)** sg(Site-3), and **(d)** sg(Site-4).

**Additional file 12: Fig. S12.** Characterization of XMAS-TREE reporter in hPSCs. Representative flow cytometry plots of hPSCs transfected pEF-XMAS-1xStop or pEF-XMAS-2xStop, pEF-ABEmax, and sg(NT) or sg(XMAS).

**Additional file 13: Fig. S13.** Comparison of editing efficiency in hPSCs at target loci using the 1xStop and 2xStop reporting vector. Quantification of base editing efficiencies at target loci in mCherry/GFP double positive cell populations using XMAS-TREE based targeting with the 1xStop or 2xStop reporting vector. Student’s t-test, N.S. = not significant. n = 3

**Additional file 14: Fig. S14.** Representative Sanger sequencing chromatographs of edited hPSCs enriched using XMAS-TREE and RoT-based approaches. Sanger sequencing chromatographs of Site-3 and PSEN1 of unsorted hPSCs as well as mCherry-positive and mCherry/GFP double positive cells isolated using RoT-based and XMAS-TREE strategies, respectively.

**Additional file 15: Fig. S15.** Distribution of genotypes in clonal hPSCs generated using XMAS-TREE-based methods. Analysis of clonal editing efficiency in hPSCs that were targeted at the PSEN1 locus.

**Additional file 16: Fig. S16.** Analysis of bystander editing in clonal hPSCs isolated using XMAS-TREE. Distribution of bystander edits in clonal hPSCs editing at genomic Site-3 (left panel) and PSEN1 (right panel).

**Additional file 17: Fig. S17.** Analysis of bystander editing in multiplex editing hPSCs isolated using XMAS-TREE. Distribution of bystander edits at target loci in mCherry/GFP double positive and unsorted hPSCs using XMAS-TREE based strategies. Orange indicates target A within the editing window. Light grey indicates bystander A within the editing window. Bystander ratio was computed as the frequency of editing the bystander A divided by the percentage of editing at the target A. *P*-value given for Student’s t-test comparing bystander ratio in mCherry/GFP double positive versus unsorted cells at an indicated bystander A.

**Additional file 18: Table S1.** List of sgRNA sequences used in this study.

**Additional file 19: Table S2.** List of primer sequences used in this study.

**Additional file 20: Table S3.** Phire PCR conditions for each target site analyzed by Sanger sequencing.

**Additional file 21: Table S4.** Parameters for EditR analysis.

**Additional file 22: Table S5.** List of primers used in this study to amplify off-target sites.

**Additional file 23.**

## Data Availability

All data generated or analyzed during this study are included in this published article and its supplementary information files. Supporting data values have also been included in Additional File 23: Table S6.
