# Supplementary material for: A Cas9-mediated adenosine transient reporter enables enrichment of ABE-targeted cells
Source: BMC Biol. 2020 Dec 14;18:193. doi: 10.1186/s12915-020-00929-7 (PMC7737295; doi:10.1186/s12915-020-00929-7)
Supplement: Supplementary file 1 — Additional file 1: Fig. S1. Transfection efficiency is not predictive of editing efficiency. HEK293 cells were transfected with pEF-mCherry, pCMV-ABEmax, and sg(TS). Comparison of transfection efficiency (percentage of mCherry-positive cells) and editing efficiency (percentage of A-to-G conversion at target nucleotides) in unsorted cell populations targeted at various genomic loci. [file 12915_2020_929_MOESM1_ESM.pdf]

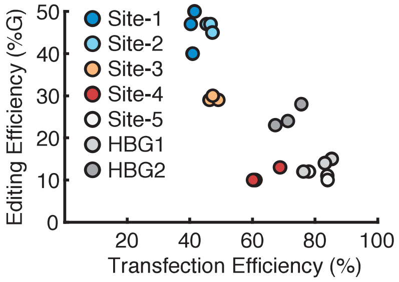

Supplemental Figure 1. Transfection efficiency is not predictive of editing efficiency. HEK293 cells were transfected with pEF-mCherry, pCMV-ABEmax, and sg(TS). Comparison of transfection efficiency (percentage of mCherry-positive cells) and editing efficiency (percentage of A-to-G conversion at target nucleotides) in unsorted cell populations targeted at various genomic loci.
