# Supplementary material for: A Cas9-mediated adenosine transient reporter enables enrichment of ABE-targeted cells
Source: BMC Biol. 2020 Dec 14;18:193. doi: 10.1186/s12915-020-00929-7 (PMC7737295; doi:10.1186/s12915-020-00929-7)
Supplement: Supplementary file 2 — Additional file 2: Fig. S2. Flow cytometry-based characterization of XMAS-TREE reporter. Representative flow cytometry plots of HEK293 cells transfected with pEF-XMAS-1xStop or pEF-XMAS-2xStop, pCMV-ABEmax, and sg(NT) or sg(XMAS). [file 12915_2020_929_MOESM2_ESM.pdf]

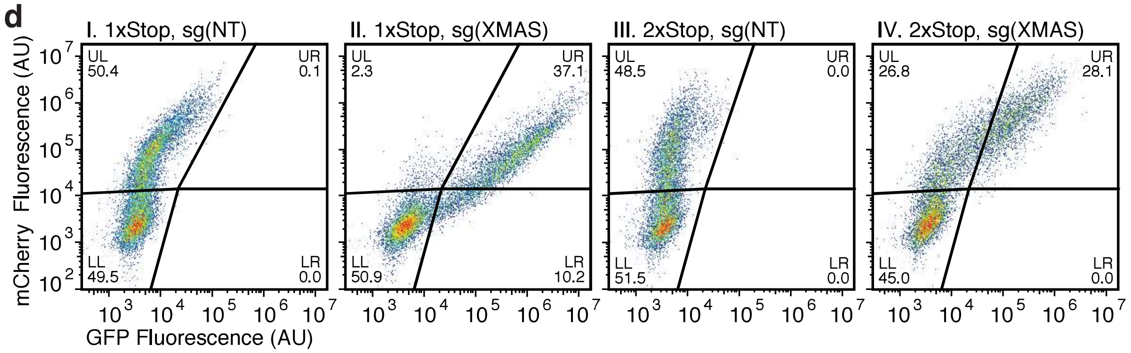

Supplemental Figure 2. Flow cytometry-based characterization of XMAS-TREE reporter. Representative flow cytometry plots of HEK293 cells transfected with pEF-XMAS-1xStop or pEF-XMAS-2xStop, pCMV-ABEmax, and sg(NT) or sg(XMAS).
