# Supplementary material for: A Cas9-mediated adenosine transient reporter enables enrichment of ABE-targeted cells
Source: BMC Biol. 2020 Dec 14;18:193. doi: 10.1186/s12915-020-00929-7 (PMC7737295; doi:10.1186/s12915-020-00929-7)
Supplement: Supplementary file 4 — Additional file 4: Fig. S4. Comparison of editing efficiency in HEK293 cells at target loci using the 1xStop and 2xStop reporting vector. Quantification of base editing efficiencies at target loci in mCherry/GFP double positive cell populations using XMAS-TREE based targeting with the 1xStop or 2xStop reporting vector. Student’s t-test, N.S. = not significant. n = 3 [file 12915_2020_929_MOESM4_ESM.pdf]

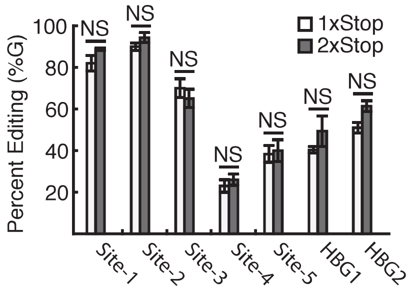

Supplemental Figure 4. Comparison of editing efficiency in HEK293 cells at target loci using the 1xStop and 2xStop reporting vector. Quantification of of base editing efficiencies at target loci in mCherry-positive/GFP-positive cell populations using XMAS-TREE based targeting with the 1xStop or 2xStop reporting vector. Student's t-test, N.S. = not significant.
