# Supplementary material for: A Cas9-mediated adenosine transient reporter enables enrichment of ABE-targeted cells
Source: BMC Biol. 2020 Dec 14;18:193. doi: 10.1186/s12915-020-00929-7 (PMC7737295; doi:10.1186/s12915-020-00929-7)
Supplement: Supplementary file 5 — Additional file 5: Fig. S5. Analysis of bystander editing in base-edited HEK293 cell populations using XMAS-TREE. Distribution of bystander edits at target loci in mCherry/GFP double positive cell populations isolated using XMAS-TREE based strategies. Orange indicates target A within the editing window. Light grey indicates bystander A within the editing window. [file 12915_2020_929_MOESM5_ESM.pdf]

Position:	20	19	18	17	16	15	14	13	12
**Site-1:**	G	A	A	C	A	C	A	A	A
**1X-Stop:**		0±0	4.3±1.5		82±3.6		23.3±1.2	1±1.07	
**2X-Stop:**		1.8±1	3±2.2		88.7±0.6		21.5±1.7	0±0	
**Site-2**	G	A	G	T	A	T	G	A	G
**1X-Stop:**		3±0			90±1.7			5±2	
**2X-Stop:**		3.8±1.5			94.3±2.3			6.25±2.63	
**Site-3**	G	A	T	G	A	G	A	T	A
**1X-Stop:**		8.8±4.0			70±4.6		67.3±8		18±2.7
**2X-Stop:**		16±2.6			65±4.4		61±3.9		24+3.9
**Site-4**	G	G	A	T	T	G	A	C	C
**1X-Stop:**			3±0				23±3		
**2X-Stop:**			2.8±0.5				26±2.6		
**Site-5**	G	T	A	G	A	A	A	A	A
**1X-Stop:**			6.5±0.7		41±2	38.3±4.0	15.7±5.7	5.3±2.5	
**2X-Stop:**			6.8±2.2		42±7.4	40±5.2	12.8±2.6	2.8±3.4	

Supplemental Figure 5. Analysis of bystander editing in base-edited HEK293 cell populations using XMAS-TREE. Distribution of bystander edits at target loci in mCherry-positive/ GFP-positive cell populations isolated using XMAS-TREE based strategies. Orange indicates target A within the editing window. Light grey indicates bystander A within the editing window.
