# Supplementary material for: A Cas9-mediated adenosine transient reporter enables enrichment of ABE-targeted cells
Source: BMC Biol. 2020 Dec 14;18:193. doi: 10.1186/s12915-020-00929-7 (PMC7737295; doi:10.1186/s12915-020-00929-7)
Supplement: Supplementary file 6 — Additional file 6: Fig. S6. Comparison of bystander editing in mCherry/GFP double positive and unsorted HEK293 cell populations using XMAS-TREE. Distribution of bystander edits at target loci in mCherry/GFP double positive and unsorted cell populations using XMAS-TREE based strategies in the context of (a) singleplex and (b) multiplex editing. Orange indicates target A within the editing window. Light grey indicates bystander A within the editing window. Bystander ratio was computed as the frequency of editing the bystander A divided by the percentage of editing at the target A. P-value given for Student’s t-test comparing bystander ratio in mCherry/GFP double positive versus unsorted cells at an indicated bystander A. n = 3 [file 12915_2020_929_MOESM6_ESM.pdf]

a

	Position:	20	19	18	17	16	15	14	13	12
**1xStop Reporter**	**Site-3**	G	A	T	G	A	G	A	T	A
**Unsorted**	**Efficiency:**		7.8±1.0			31.8±1.5		30±1.4		12±1.4
	**Bystander ratio:**		0.24±0.02			N.A.		0.94±0.02		0.47±0.04
**mCherry+, GFP+**	**Efficiency:**		8.8±4.0			67.3±8.0		66.8±7.5		18±2.7
	**Bystander ratio:**		0.13±0.07			N.A.		0.99±0.01		0.27±0.08
	**P-VALUE**		**0.09**			N.A.		**0.01**		**0.01**
**2xStop Reporter**										
**Unsorted**	**Efficiency:**		10.5±0.6			25.8±3.3		25.3±4.1		17.5±1.3
	**Bystander ratio:**		0.42±0.07			N.A.		0.98±0.02		0.70±0.16
**mCherry+, GFP+**	**Efficiency:**		16±2.6			61±3.9		62±5.7		24±3.9
	**Bystander ratio:**		0.26±0.06			N.A.		1.02±0.03		0.40±0.09
	**P-VALUE**		**0.03**			N.A.		**0.07**		**0.03**
**1xStop Reporter**	**HBG1**	C	T	T	G	A	C	C	A	A
**Unsorted**	**Efficiency:**					15±2.9			10±2.6	2.5±5.8
	**Bystander ratio:**					N.A.			0.66±0.06	0.17±0.05
**mCherry+, GFP+**	**Efficiency:**					38.8±3.4			26.5±1.7	5.8±2.2
	**Bystander ratio:**					N.A.			0.68±0.06	0.15±0.07
	**P-VALUE**					N.A.			**0.62**	**0.76**
**2xStop Reporter**										
**Unsorted**	**Efficiency:**					12.5±3.4			9.25±2.2	1.8±0.5
	**Bystander ratio:**					N.A.			0.74±0.03	0.14±0.3
**mCherry+, GFP+**	**Efficiency:**					47±7.5			33.5±11.3	5±1.8
	**Bystander ratio:**					N.A.			0.71±0.18	0.11±0.05
	**P-VALUE**					N.A.			**0.73**	**0.44**
**1xStop Reporter**	**HBG2**	A	T	A	T	T	T	G	C	A
**Unsorted**	**Efficiency:**			25±6.3						6.3±1.2
	**Bystander ratio:**			N.A.						0.25±0.048
**mCherry+, GFP+**	**Efficiency:**			51±2.6						12.3±4.0
	**Bystander ratio:**			N.A.						0.24±0.08
	**P-VALUE**			N.A.						**0.86**
**2xStop Reporter**										
**Unsorted**	**Efficiency:**			22.5±2.6						6.8±2.2
	**Bystander ratio:**			N.A.						0.30±0.08
**mCherry+, GFP+**	**Efficiency:**			59.5±4.2						12.8±3.1
	**Bystander ratio:**			N.A.						0.21±0.05
	**P-VALUE**			N.A						**0.27**

b

	Position:	20	19	18	17	16	15	14	13	12
**1xStop Reporter**	**Site-1:**	G	A	A	C	A	C	A	A	A
**Unsorted**	**Efficiency:**		N.D.	N.D.		55.25±4.8		21±7.7	N.D.	
	**Bystander ratio:**		N.D.	N.D.		N.A.		0.38±0.01	N.D.	
**mCherry+, GFP+**	**Efficiency:**		N.D.	N.D.		77±4.8		26.3±7.7	N.D.	
	**Bystander ratio:**		N.D.	N.D.		N.A.		0.34±0.11	N.D.	
	**P-VALUE**		N.A.	N.A.		N.A.		**0.55**	N.A.	
**1xStop Reporter**	**Site-3**	G	A	T	G	A	G	A	T	A
**Unsorted**	**Efficiency:**		2±1			29±1.7		28.3±2.1		9±0
	**Bystander ratio:**		0.07±0.03			N.A.		1.01±0.07		0.31±0.02
**mCherry+, GFP+**	**Efficiency:**		8.7±7.3			69.3±4.7		73.7±8.7		21±8
	**Bystander ratio:**		0.01±0.1			N.A.		1.06±0.06		0.31±0.14
	**P-VALUE**		**0.53**			N.A.		**0.18**		**0.98**
**1xStop Reporter**	**Site-4**	G	G	A	T	T	G	A	C	C
**Unsorted**	**Efficiency:**			4±1.7				17.3±0.6		
	**Bystander ratio:**			0.23±0.11				N.A.		
**mCherry+, GFP+**	**Efficiency:**			3.3±1.5				26.7±11.5		
	**Bystander ratio:**			0.13±0.01				N.A.		
	**P-VALUE**			**0.18**				N.A.		
	**Position:**	20	19	18	17	16	15	14	13	12
**2xStop Reporter**	**Site-5**	G	T	A	G	A	A	A	A	A
**Unsorted**	**Efficiency:**			4.5±1.3		26.75±3.3	19.8±2.1	8.5±1	2.5±1	
	**Bystander ratio:**			0.23±0.05		1.35±0.07	N.A.	0.43±0.04	0.13±0.05	
**mCherry+, GFP+**	**Efficiency:**			9.3±1		79.25±3.3	67.3±3.2	25.3±6.8	5.3±3.3	
	**Bystander ratio:**			0.13±0.01		1.18±0.07		0.38±0.11	0.08±0.05	
	**P-VALUE**			**0.03**		**0.045**		0.43	0.34	
**2xStop Reporter**	**HBG1**	C	T	T	G	A	C	C	A	A
**Unsorted**	**Efficiency:**					9.5±0.6			14.75±1.7	6.75±1.5
	**Bystander ratio:**					N.A.			1.55±0.11	0.71±0.16
**mCherry+, GFP+**	**Efficiency:**					33±3.3			34±1.5	16.5±4.8
	**Bystander ratio:**					N.A.			1.04±0.07	0.49±0.13
	**P-VALUE**					N.A.			**0.001**	**0.22**
**2xStop Reporter**	**HBG1**	A	T	A	T	T	T	G	C	A
**Unsorted**	**Efficiency:**			19.3±0.5						9.25±1
	**Bystander ratio:**			N.A.						0.48±0.05
**mCherry+, GFP+**	**Efficiency:**			50±7.6						23±0.62
	**Bystander ratio:**			N.A.						0.47±0.07
	**P-VALUE**			N.A.						**0.62**

Supplemental figure 6: Comparison of bystander editing in mCherry/GFP double positive and unsorted HEK293 cell populations using XMAS-TREE. (caption on next page)

Supplemental Figure 6. Comparison of bystander editing in mCherry/GFP double positive and unsorted HEK293 cell populations using XMAS-TREE. Distribution of bystander edits at target loci in mCherry/GFP double positive and unsorted cell populations using XMAS-TREE based strategies in the context of (**a**) singleplex and (**b**) multiplex editing. Orange indicates target A within the editing window. Light grey indicates bystander A within the editing window. Bystander ratio was computed as the frequency of editing the bystander A divided by the percentage of editing at the target A. P-value given for Student's t-test comparing bystander ratio in mCherry/GFP double positive versus unsorted cells at an indicated bystander A.
