# Supplementary material for: A Cas9-mediated adenosine transient reporter enables enrichment of ABE-targeted cells
Source: BMC Biol. 2020 Dec 14;18:193. doi: 10.1186/s12915-020-00929-7 (PMC7737295; doi:10.1186/s12915-020-00929-7)
Supplement: Supplementary file 7 — Additional file 7: Fig. S7. Comparison of XMAS-TREE editing efficiency in individual- or multiplexed-targeted genomic sites in HEK293 cells. Quantification of base editing efficiencies at targeted loci in mCherry/GFP double positive cell populations using XMAS-TREE-based targeting in a single or multiplexed manner. Student’s t-test; N.S. = not significant, * = p < 0.05. n = 3 [file 12915_2020_929_MOESM7_ESM.pdf]

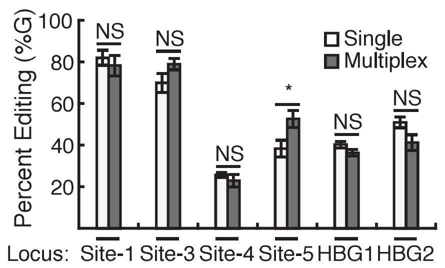

Supplemental Figure 7. Comparison of XMAS-TREE editing efficiency in individual- or multiplexed-targeted genomic sites in HEK293 cells. Quantification of base editing efficiencies at targeted loci in mCherry-positive/GFP-positive cell populations using XMAS-TREE-based targeting in a single or multiplexed manner. Student's t-test; N.S. = not significant, * = $p<0.05$.
