# Supplementary material for: A Cas9-mediated adenosine transient reporter enables enrichment of ABE-targeted cells
Source: BMC Biol. 2020 Dec 14;18:193. doi: 10.1186/s12915-020-00929-7 (PMC7737295; doi:10.1186/s12915-020-00929-7)
Supplement: Supplementary file 8 — Additional file 8: Fig. S8. Comparison of multiplexed editing efficiency in HEK293 cells isolated using RoT and XMAS-TREE approaches. Quantification of multiplexed base editing efficiencies at target loci in mCherry-positive cells isolated using RoT and mCherry-positive/GFP-positive cells isolated using XMAS-TREE. Student’s t-test; N.S. = not significant, * = p < 0.05, ** = p < 0.01. n = 3 [file 12915_2020_929_MOESM8_ESM.pdf]

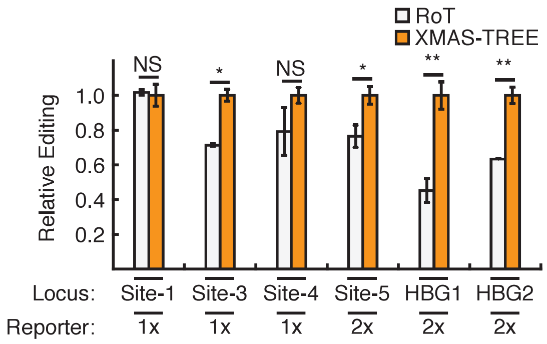

Supplemental Figure 8. Comparison of multiplexed editing efficiency in HEK293 cells isolated using RoT and XMAS-Tree approaches. Quantification of mutliplexed base editing efficiencies at target loci in mCherry-positive cells isolated using RoT and mCherry-positive/GFPpositvie cells isolated using XMAS-TREE. Student's t-test; N.S. = not significant, * = $p<0.05$, ** = $p<0.01$.
