# Supplementary material for: A Cas9-mediated adenosine transient reporter enables enrichment of ABE-targeted cells
Source: BMC Biol. 2020 Dec 14;18:193. doi: 10.1186/s12915-020-00929-7 (PMC7737295; doi:10.1186/s12915-020-00929-7)
Supplement: Supplementary file 9 — Additional file 9: Fig. S9. Representative Sanger sequences from clonal HEK293 cells isolated using XMAS-TREE in a multiplexed manner. Sanger sequences from a representative clone isolated using XMAS-TREE that had homozygous edits at all three-target loci, Site-1/Site-3/Site-4. [file 12915_2020_929_MOESM9_ESM.pdf]

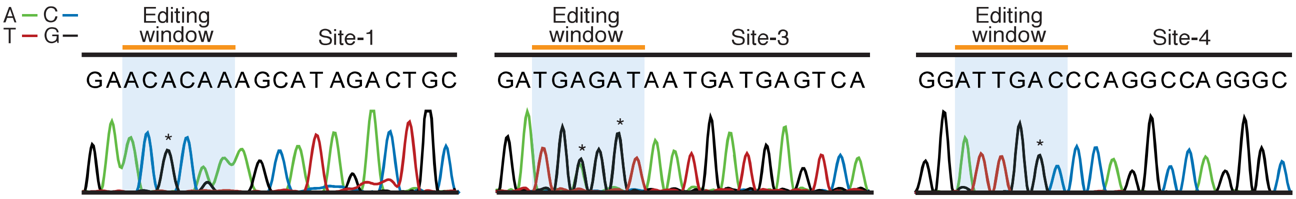

Supplemental Figure 9. Representative Sanger sequences from clonal HEK293 cells isolated using XMAS-TREE in a multiplexed manner. Sanger sequences from a representative clone isolated using XMAS-TREE that had homozygous edits at all three-target loci, Site-1/Site-3/Site-4.
