# Supplementary material for: A Cas9-mediated adenosine transient reporter enables enrichment of ABE-targeted cells
Source: BMC Biol. 2020 Dec 14;18:193. doi: 10.1186/s12915-020-00929-7 (PMC7737295; doi:10.1186/s12915-020-00929-7)
Supplement: Supplementary file 10 — Additional file 10: Fig. S10. Analysis of bystander editing in clonal multiplexed base-edited HEK293 cell populations using XMAS-TREE. Distribution of bystander edits at genomic Site-1/Site-3/Site-4 in clonal HEK293 cells that were generated via multiplexed base editing. [file 12915_2020_929_MOESM10_ESM.pdf]

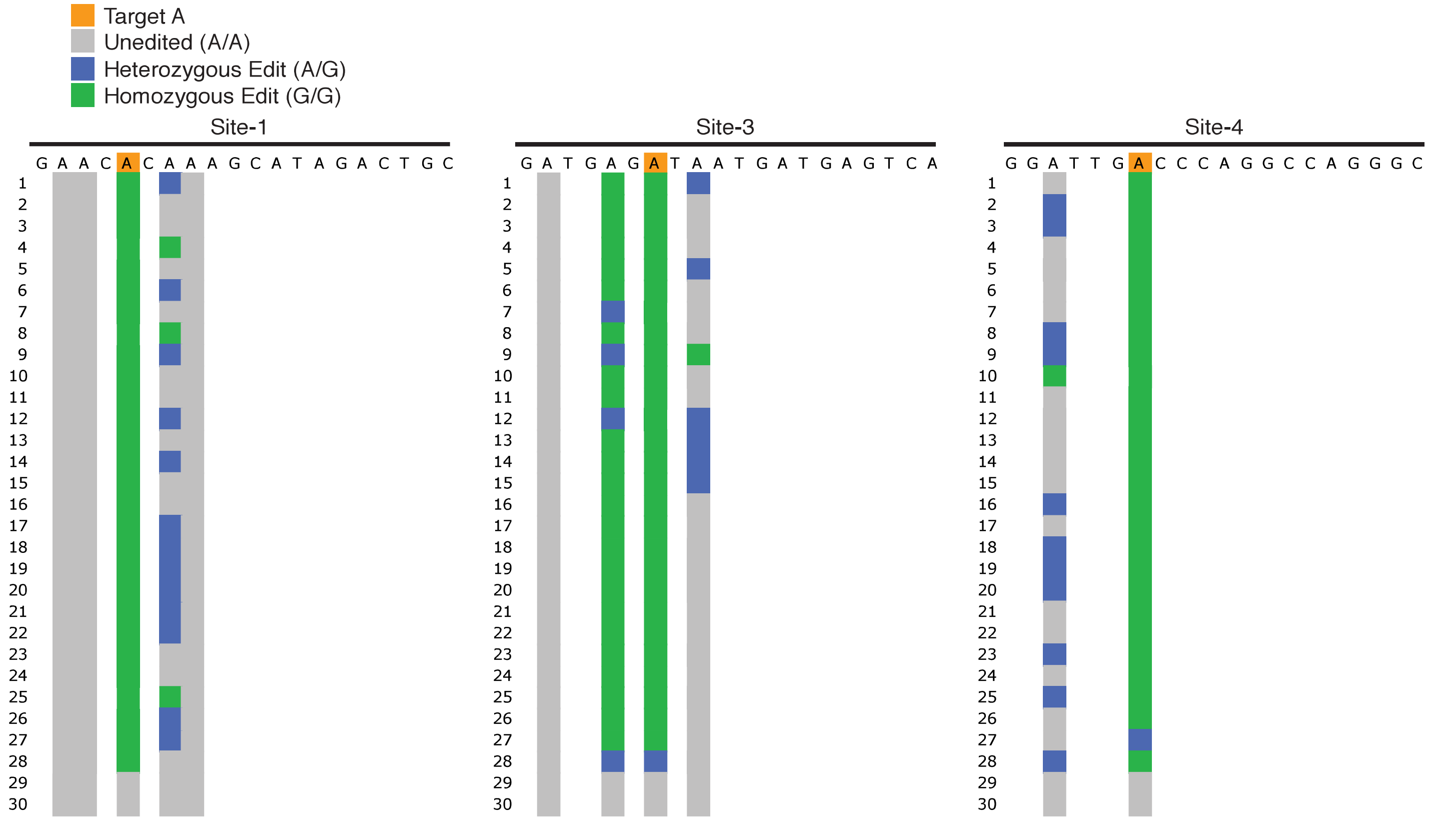

Supplemental Figure 10. Analysis of bystander editing in clonal multiplexed base-edited HEK293 cell populations using XMAS-TREE. Distribution of bystander edits at genomic Site-1/Site-3/Site-4 in clonal HEK293 cells that were generated via multiplexed base editing.
