# Supplementary material for: A Cas9-mediated adenosine transient reporter enables enrichment of ABE-targeted cells
Source: BMC Biol. 2020 Dec 14;18:193. doi: 10.1186/s12915-020-00929-7 (PMC7737295; doi:10.1186/s12915-020-00929-7)
Supplement: Supplementary file 11 — Additional file 11: Fig. S11. Off-target analysis of HEK293 clones generated using XMAS-TREE-based methods. Representative clonal lines were analyzed by Sanger sequencing at the top predicted off-target loci for the sgRNA sequences for (a) sg(XMAS), (b) sg(Site-1), (c) sg(Site-3), and (d) sg(Site-4). [file 12915_2020_929_MOESM11_ESM.pdf]

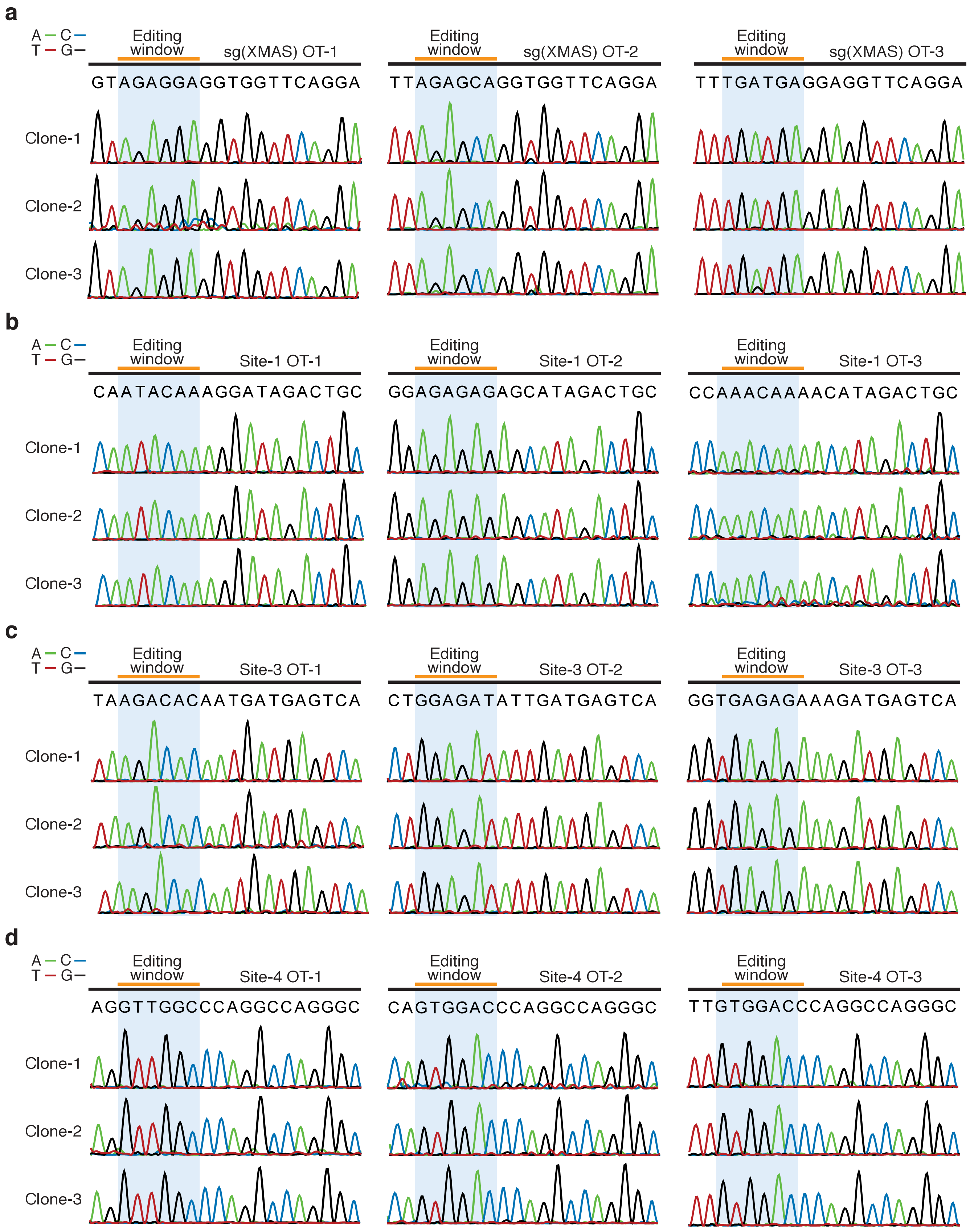

Supplemental Figure 11. Off-target analysis of HEK293 clones generated using XMASTREE-based methods. Representative clonal lines were analyzed by Sanger sequencing at the top predicted off-target loci for the sgRNA sequences for (**a**) sg(XMAS), (**b**) sg(Site-1), (**c**) sg(Site-3), and (**d**) sg(Site-4).
