# Supplementary material for: A Cas9-mediated adenosine transient reporter enables enrichment of ABE-targeted cells
Source: BMC Biol. 2020 Dec 14;18:193. doi: 10.1186/s12915-020-00929-7 (PMC7737295; doi:10.1186/s12915-020-00929-7)
Supplement: Supplementary file 13 — Additional file 13: Fig. S13. Comparison of editing efficiency in hPSCs at target loci using the 1xStop and 2xStop reporting vector. Quantification of base editing efficiencies at target loci in mCherry/GFP double positive cell populations using XMAS-TREE based targeting with the 1xStop or 2xStop reporting vector. Student’s t-test, N.S. = not significant. n = 3 [file 12915_2020_929_MOESM13_ESM.pdf]

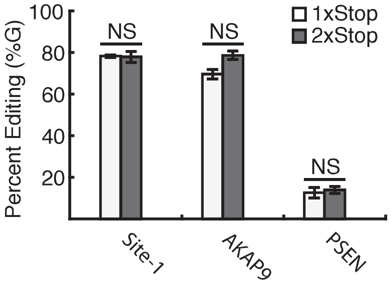

Supplemental Figure 13. Comparison of editing efficiency in hPSCs at target loci using the 1xStop and 2xStop reporting vector. Quantification of base editing efficiencies at target loci in mCherry-positive/GFP-positive cell populations using XMAS-TREE based targeting with the 1xStop or 2xStop reporting vector. Student's t-test, N.S. = not significant.
