# Supplementary material for: A Cas9-mediated adenosine transient reporter enables enrichment of ABE-targeted cells
Source: BMC Biol. 2020 Dec 14;18:193. doi: 10.1186/s12915-020-00929-7 (PMC7737295; doi:10.1186/s12915-020-00929-7)
Supplement: Supplementary file 14 — Additional file 14: Fig. S14. Representative Sanger sequencing chromatographs of edited hPSCs enriched using XMAS-TREE and RoT-based approaches. Sanger sequencing chromatographs of Site-3 and PSEN1 of unsorted hPSCs as well as mCherry-positive and mCherry/GFP double positive cells isolated using RoT-based and XMAS-TREE strategies, respectively. [file 12915_2020_929_MOESM14_ESM.pdf]

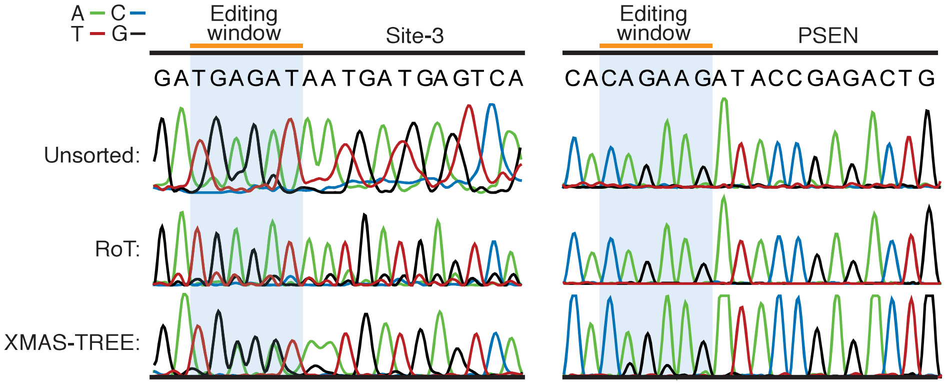

Supplemental Figure 14. Representative Sanger sequencing chromatographs of edited hPSCs enriched using XMAS-TREE and RoT-based approaches. Sanger sequencing chromatographs of Site-3 and PSEN1 of unsorted hPSCs as well as mCherry-positive and mCherry-positive/GFP-positive cells isolated using RoT-based and XMAS-TREE strategies, respectively.
