# Supplementary material for: A Cas9-mediated adenosine transient reporter enables enrichment of ABE-targeted cells
Source: BMC Biol. 2020 Dec 14;18:193. doi: 10.1186/s12915-020-00929-7 (PMC7737295; doi:10.1186/s12915-020-00929-7)
Supplement: Supplementary file 15 — Additional file 15: Fig. S15. Distribution of genotypes in clonal hPSCs generated using XMAS-TREE-based methods. Analysis of clonal editing efficiency in hPSCs that were targeted at the PSEN1 locus. [file 12915_2020_929_MOESM15_ESM.pdf]

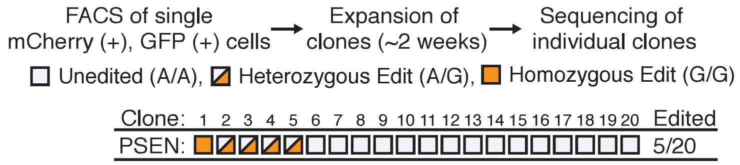

Supplemental Figure 15. Distribution of genotypes in clonal hPSCs generated using XMAS-TREE- based methods. Analysis of clonal editing efficiency in hPSCs that were targeted at the PSEN1 locus.
