# Supplementary material for: A Cas9-mediated adenosine transient reporter enables enrichment of ABE-targeted cells
Source: BMC Biol. 2020 Dec 14;18:193. doi: 10.1186/s12915-020-00929-7 (PMC7737295; doi:10.1186/s12915-020-00929-7)
Supplement: Supplementary file 16 — Additional file 16: Fig. S16. Analysis of bystander editing in clonal hPSCs isolated using XMAS-TREE. Distribution of bystander edits in clonal hPSCs editing at genomic Site-3 (left panel) and PSEN1 (right panel). [file 12915_2020_929_MOESM16_ESM.pdf]

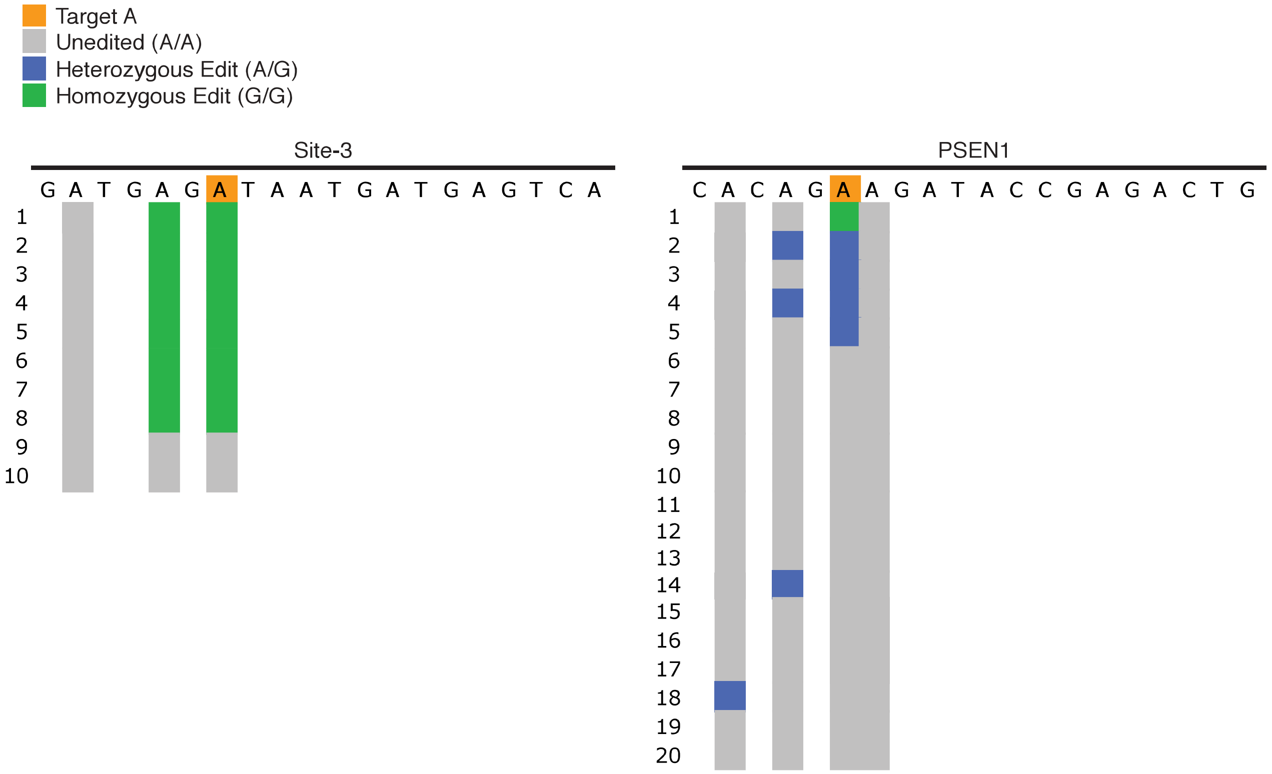

Supplemental Figure 16. Analysis of bystander editing in clonal hPSCs isolated using XMAS-TREE. Distribution of bystander edits in clonal hPSCs editing at genomic Site-3 (left panel) and PSEN1 (right panel).
