# Supplementary material for: A Cas9-mediated adenosine transient reporter enables enrichment of ABE-targeted cells
Source: BMC Biol. 2020 Dec 14;18:193. doi: 10.1186/s12915-020-00929-7 (PMC7737295; doi:10.1186/s12915-020-00929-7)
Supplement: Supplementary file 17 — Additional file 17: Fig. S17. Analysis of bystander editing in multiplex editing hPSCs isolated using XMAS-TREE. Distribution of bystander edits at target loci in mCherry/GFP double positive and unsorted hPSCs using XMAS-TREE based strategies. Orange indicates target A within the editing window. Light grey indicates bystander A within the editing window. Bystander ratio was computed as the frequency of editing the bystander A divided by the percentage of editing at the target A. P-value given for Student’s t-test comparing bystander ratio in mCherry/GFP double positive versus unsorted cells at an indicated bystander A. [file 12915_2020_929_MOESM17_ESM.pdf]

		20	19	18	17	16	15	14	13	12
	**Site-5**	G	T	A	G	A	A	A	A	A
**Unsorted**	**Efficiency:**			1±0		6.7±0.6	4.3±1.2	1.3±1.5	0.3±0.6	N.D.
	**Bystander ratio:**			0.24±0.1		1.6±0.35	N.A.	0.27±0.31	0.11±0.19	N.D.
**mCherry+, GFP+**	**Efficiency:**			9±5.2		79.7±5.1	52.7±4.0	14±2.1	7.7±5.9	N.D.
	**Bystander ratio:**			0.14±0.19		1.52±0.17	N.A.	0.27±0.17	0.14±0.10	N.D.
	**P-VALUE**			**0.56**		**0.79**	N.A.	**0.98**	**0.79**	N.A.
	**HBG1**	C	T	T	G	A	C	C	A	A
**Unsorted**	**Efficiency:**					4.6±0.6			2.3±0.6	0±0
	**Bystander ratio:**					N.A.			0.52±0.2	0±0
**mCherry+, GFP+**	**Efficiency:**					22.7±1.5			20.3±4.0	10±2.6
	**Bystander ratio:**					N.A.			0.90±0.21	0.4±0.11
	**P-VALUE**					N.A.			**0.06**	**0.02**
	**HBG2**	A	T	A	T	T	T	G	C	A
**Unsorted**	**Efficiency:**			2±1.7						3±1.7
	**Bystander ratio:**			N.A.						0.56±0.69
**mCherry+, GFP+**	**Efficiency:**			41.3±3.8						6±3
	**Bystander ratio:**			N.A.						0.15±0.09
	**P-VALUE**			N.A.						**0.41**

Supplemental Figure 17. Analysis of bystander editing in multiplex editing hPSCs isolated using XMAS-TREE. Distribution of bystander edits at target loci in mCherry/GFP double positive and unsorted hPSCs using XMAS-TREE based strategies. Orange indicates target A within the editing window. Light grey indicates bystander A within the editing window. Bystander ratio was computed as the frequency of editing the bystander A divided by the percentage of editing at the target A. P-value given for Student's t-test comparing bystander ratio in mCherry/GFP double positive versus unsorted cells at an indicated bystander A.
