# Supplementary material for: A Cas9-mediated adenosine transient reporter enables enrichment of ABE-targeted cells
Source: BMC Biol. 2020 Dec 14;18:193. doi: 10.1186/s12915-020-00929-7 (PMC7737295; doi:10.1186/s12915-020-00929-7)
Supplement: Supplementary file 18 — Additional file 18: Table S1. List of sgRNA sequences used in this study. [file 12915_2020_929_MOESM18_ESM.pdf]

SUPPLEMENTAL TABLES

Additional File 18: Table S1. List of sgRNA sequences used in this study.

Site	Sequence (5'→3')
XMAS-1xStop	GTTGATGGGGTGGTTCAGGA
XMAS-2xStop	GTTGATGAGGTGGTTCAGGA
Site-1	GAACACAAAGCATAGACTGC
Site-2	GAGTATGAGGCATAGACTGC
Site-3	GATGAGATAATGATGAGTCA
Site-4	GGATTGACCCAGGCCAGGGC
Site-5	GTAGAAAAAGTATAGACTGC
HBG1	GCTTGACCAATAGCCTTGACA
HBG2	GATATTTGCATTGAGATAGTG
AKAP9	GAAAATAGTTGAAGAAAAAG
PSEN	GCACAGAAGATACCGAGACTG
Non-target (NT)	GGGTCTTCGAGAAGACCT
