# Supplementary material for: A Cas9-mediated adenosine transient reporter enables enrichment of ABE-targeted cells
Source: BMC Biol. 2020 Dec 14;18:193. doi: 10.1186/s12915-020-00929-7 (PMC7737295; doi:10.1186/s12915-020-00929-7)
Supplement: Supplementary file 19 — Additional file 19: Table S2. List of primer sequences used in this study. [file 12915_2020_929_MOESM19_ESM.pdf]

Additional File 19: Table S2. List of primer sequences used in this study.

Primer	Forward Sequence (5'→3')	Reverse Sequence (5'→3')
Site-1	TCCTTGGAAACAATGATAACAAGAC	CCAGCCCCATCTGTCAAACT
Site-2	GCTTATATTCTAGGGAGACAGACAT	ACCTGAGGTCAGAAGTTTGAGA
Site-3	GTCTGAGGTCACACAGTGGG	AGAGCAGGGACCACATCTAC
Site-4	GCCAAACTTGTCAACCAGTA	ATGTGGGCTGCCTAGAAAGG
Site-5	TCCATTTATATGAAATGTTCAGAAAAGGCAAAT	GTAACTATATGCTCTCTGATTCTCCTATTAGC
HBG1	CCTACCTTCCCAGGGTTT	AAGAAGTCCTGGTATCTTCTATG
HBG2	TCAGACGTTCCAGAAGCGAG	GACAAGAAGGTGAAAAACGGCTG
AKAP9	GATTCAAAGCATACCAGAGAATAGT	TCAAACTAGTATGCATTTCAACAAC
PSEN1	GAGTGTAGCTGTTTTTCTCAGGTT	GAATACCCAACCATAAGAAGAACAG
