# Supplementary material for: A Cas9-mediated adenosine transient reporter enables enrichment of ABE-targeted cells
Source: BMC Biol. 2020 Dec 14;18:193. doi: 10.1186/s12915-020-00929-7 (PMC7737295; doi:10.1186/s12915-020-00929-7)
Supplement: Supplementary file 20 — Additional file 20: Table S3. Phire PCR conditions for each target site analyzed by Sanger sequencing. [file 12915_2020_929_MOESM20_ESM.pdf]

Additional File 20: Table S3. Phire PCR conditions for each target site analyzed by Sanger sequencing.

Target	Initial denature time and temperature	Denature time and temperature	Annealing time and temperature	Extension time and temperature	Final extension time and temperature
		40 cycles			
Site-1	98 C 5 min	98 C 5 sec	56 C 5 sec	72 C 30 sec	72 C 5 min
Site-2	98 C 5 min	98 C 5 sec	62 C 5 sec	72 C 30 sec	72 C 5 min
Site-3	98 C 5 min	98 C 5 sec	56.8 C 5 sec	72 C 30 sec	72 C 5 min
Site-4	98 C 5 min	98 C 5 sec	61.3 C 5 sec	72 C 30 sec	72 C 5 min
Site-5	98 C 5 min	98 C 5 sec	65 C 5 sec	72 C 30 sec	72 C 5 min
HBG1	98 C 5 min	98 C 5 sec	59.2 C 5 sec	72 C 30 sec	72 C 5 min
HBG2	98 C 5 min	98 C 5 sec	59 C 5 sec	72 C 30 sec	72 C 5 min
AKAP9	98 C 5 min	98 C 5 sec	64 C 5 sec	72 C 30 sec	72 C 5 min
PSEN1	98 C 5 min	98 C 5 sec	63 C 5 sec	72 C 30 sec	72 C 5 min
