# Supplementary material for: A Cas9-mediated adenosine transient reporter enables enrichment of ABE-targeted cells
Source: BMC Biol. 2020 Dec 14;18:193. doi: 10.1186/s12915-020-00929-7 (PMC7737295; doi:10.1186/s12915-020-00929-7)
Supplement: Supplementary file 21 — Additional file 21: Table S4. Parameters for EditR analysis. [file 12915_2020_929_MOESM21_ESM.pdf]

Additional File 21: Table S4. Parameters for EditR analysis.

Target Site	Protospacer	5' bound	3' bound
Site-1	GAACACAAAGCATAGACTGC	50	120
Site-2	GAGTATGAGGCATAGACTGC	140	200
Site-3	GATGAGATAATGATGAGTCA	100	180
Site-4	GGATTGACCCAGGCCAGGGC	80	160
Site-5	GCAGTCTATACTTTTTCTAC	40	120
HBG1	CTTGACCAATAGCCTTGACA	160	260
HBG2	ATATTTGCATTGAGATAGTG	120	220
AKAP9	GAAAATAGTTGAAGAAAAAG	200	300
PSEN1	CACAGAAGATACCGAGACTG	120	200
