# Supplementary material for: A Cas9-mediated adenosine transient reporter enables enrichment of ABE-targeted cells
Source: BMC Biol. 2020 Dec 14;18:193. doi: 10.1186/s12915-020-00929-7 (PMC7737295; doi:10.1186/s12915-020-00929-7)
Supplement: Supplementary file 22 — Additional file 22: Table S5. List of primers used in this study to amplify off-target sites. [file 12915_2020_929_MOESM22_ESM.pdf]

Additional File 22: Table S5. List of primers used in this study to amplify off-target sites.

Primer	Forward Sequence (5'→3')	Reverse Sequence (5'→3')
XMAS-OT1	CAGCATTATCCATTTGCTGCCA	TGGAGACAGCGAGTCTACAGC
XMAS-OT2	TAACACCATTATAGCTGAAGTGGGG	TGAGTTACACACAAGCCAGTTAAATTC
XMAS-OT3	AGGGAGTGGACATGAGGCGA	CCCAAGAGGAAGTCCCAAGG
Site-1-OT1	CCTTGGGAAGAGAAGGGGTC	GAGATACCGGAAGCTTTGATGTAAGA
Site-1-OT2	CTTGGGGAGAAAGGTCCAGG	CAAGCTTTTCCTCCTGGGATGTAAAA
Site-1-OT3	CTGGCAAGCTGTTCTCACATG	GAGGCTGAGGCAGGAGTATG
Site-3-OT1	GTTTTCAGTAGAAGAGTATATAATACATAAT	ATATTCTCAGCCTAGGCCTG
Site-3-OT2	TGTTGGACATGGGTGCCTTATT	TTCACCCTCTCTGGATGGCG
Site-3-OT3	GCAGGAGGAGGCAGTGAAAG	CAGAGAAATAACACTCTGGCAGCTG
Site-4-OT1	CAGCATTTATCACGCAGTATTGTTATTG	TCATTTCGTGTTGTGCTTTATCACTTAAAA
Site-4-OT2	GTGAGCAGTAAACTTAATTGTTGATACAATAAATC	CTTTTAGAATGAAAGTGTGCATCTTAGTAAAGAAA
Site-4-OT3	GTTCCTCACTGATTCTCAGCAGG	CACAAAAGGGATAAATGCTCTATCCATTT
